# Steroid receptor coactivator-3 inhibition generates breast cancer antitumor immune microenvironment

**DOI:** 10.1186/s13058-022-01568-2

**Published:** 2022-10-31

**Authors:** Sang Jun Han, Nuri Sung, Jin Wang, Bert W. O’Malley, David M. Lonard

**Affiliations:** 1 Department of Molecular and Cellular Biology, Baylor College of Medicine, Houston, TX 77030 USA; 2 Duncan Cancer Center for Reproductive Medicine, Baylor College of Medicine, Houston, TX 77030 USA; 3 Department of Pharmacology and Chemical Biology, Baylor College of Medicine, Houston, TX 77030 USA

**Keywords:** Steroid receptor coactivator inhibitor, Antitumor immunity, Breast cancer, E0771 cells, Interleukin 1 receptor antagonist, C-X-C motif chemokine ligand 9

## Abstract

**Background:**

The tumor immune microenvironment (TIME) generated by cancer-infiltrating immune cells has a crucial role in promoting or suppressing breast cancer progression. However, whether the steroid receptor coactivator-3 (SRC-3) modulates TIME to progress breast cancer is unclear. Therefore, the present study evaluates whether SRC-3 generates a tumor-promoting TIME in breast tumors using a syngeneic immune-intact mouse model of breast cancer.

**Methods:**

We employed E0771 and 4T1 breast cancer in immune-intact syngeneic female C57BL/6 and BALB/c mice, respectively. SI-2, a specific small-molecule inhibitor of SRC-3, was administered daily (2.5 mg/kg) to E0771 and 4T1 breast tumor-bearing immune-intact mice. In addition, SRC-3 knockdown (KD)-E0771 and SRC-3 KD-4T1 cells and their parental breast cancer cells were injected into their syngeneic immune-intact female mice versus immune-deficiency mice to validate that the host immune system is required for breast tumor suppression by SRC-3 KD in immune-intact mice. Furthermore, tumor-infiltrating immune cells (such as CD4+, CD8+, CD56+, and Foxp3+ cells) in E0771 and 4T1 breast cancers treated with SI-2 and in SRC-3 KD E0771 and 4T1 breast cancers were determined by immunohistochemistry. Additionally, cytokine levels in SI-2-treated and SRC-3 KD E0771 breast tumors and their control cancers were defined with a Mouse Cytokine Array.

**Results:**

SRC-3 inhibition by SI-2 significantly suppressed the progression of breast cancer cells (E0771 and 4T1) into breast cancers in immune-intact syngeneic female mice. SRC-3 KD-E0771 and -4T1 breast cancer cells did not produce well-developed tumors in immune-intact syngeneic female mice compared to their parental cells, but SRC-3 KD breast cancers were well developed in immune-defective host mice. SRC-3 inhibition by SI-2 and SRC-3 KD effectively increased the numbers of cytotoxic immune cells, such as CD4+ and CD8+ T cells and CD56+ NK cells, and Interferon γ (Ifng) in breast cancers compared to vehicle. However, SI-2 treatment reduced the number of tumor-infiltrating CD4+/Foxp3+ regulatory T (Treg) cells compared to vehicle treatment. In addition, SRC-3 inhibition by SI-2 and SRC-3 KD increased C-X-C motif chemokine ligand 9 (Cxcl9) expression in breast cancer to recruit C-X-C motif chemokine receptor 3 (Cxcr3)-expressing cytotoxic immune cells into breast tumors.

**Conclusions:**

SRC-3 is a critical immunomodulator in breast cancer, generating a protumor immune microenvironment. SRC-3 inhibition by SI-2 or SRC-3 KD activates the Cxcl9/Cxcr3 axis in breast tumors and enhances the antitumor immune microenvironment to suppress breast cancer progression.

**Supplementary Information:**

The online version contains supplementary material available at 10.1186/s13058-022-01568-2.

## Background

Steroid receptor coactivator (SRC) knockout mouse models have revealed essential roles for SRCs in modulating multiple cellular pathways [1, 2]. SRC levels are precisely regulated in a tissue-dependent manner, and their dysregulation may lead to various human diseases, including cancer [3]. SRC-3 is more frequently reported and extensively studied in breast cancer than the other two SRC family members. The SRC-3 gene is amplified in 5–10% of breast cancers [4, 5], and the levels of the SRC-3 mRNA or protein are increased by ~ 60% in multiple different cohorts of patients with breast cancer [6, 7]. Additionally, clinical data indicate that SRC-3 overexpression is associated with more aggressive breast cancers and poorer survival rates [5, 8, 9]. In addition to exhibiting elevated expression levels, SRC-3 is considered a master regulator of breast cancer progression because it is located at the nexus of multiple oncogenic pathways, simultaneously driving proliferation, migration, invasion, and metastasis [10]. These observations imply that SRC-3 is a critical molecular therapeutic target for effectively suppressing breast cancer progression.

To effectively target SRC-3, we developed a highly potent and specific small-molecule inhibitor of SRC-3 named SI-2 that selectively inhibits the transcriptional activity of SRC-3 and reduces the SRC-3 protein concentration in cells through a direct physical interaction [11]. As a result, SI-2 selectively suppresses the proliferation of breast cancer cells at concentrations in the low nanomolar range (IC_50_ of 3–20 nM) but does not substantially affect the viability of normal cells due to the exceptional dependence of cancer cells on SRC-3 [11]. In addition to its effects in vitro*,* SI-2 (2 mg/kg) treatment also effectively suppresses the growth of human MDA-MB-468 breast tumors in a severe combined immunodeficiency (SCID) mouse xenograft model in vivo without causing significant toxicity in the heart or other major organs [11].

Tumor-infiltrating immune cells promote or suppress cancer growth during tumor progression by communicating with primary tumor cells [12]. For example, breast cancer cells produce chemokines [such as chemokine (C–C motif) ligand (CCL)5 and monocyte chemoattractant protein-1 (MCP-1)] and recruit monocytic cells that then differentiate into protumorigenic macrophages in the presence of interleukin **(**IL)-4 secreted by type 2 T helper cells to enhance their growth [13]. Breast tumors also recruit regulatory T (Treg) cells by secreting CCL22, contributing to establishing an immunosuppressive tumor environment [14]. In contrast, other chemokines produced by breast cancer cells, such as C-X-C motif chemokine ligand (CXCL)16, promote the recruitment of C-X-C chemokine receptor (CXCR)6 + antitumor CD8 + T cells to suppress breast tumor progression [15]. Characterizing alterations in the tumor-infiltrating immune cell repertoire thus provides essential insights for understanding the tumor immune microenvironment (TIME) and developing new cancer immunotherapeutics. Our recent studies revealed a critical role for SRC-3 in the function of Tregs [16]. In addition to affecting the primary tumor, SRC-3 might modulate Treg function to generate protumorigenic immune cells that enhance breast cancer progression. These observations suggest that SI-2-mediated targeting of SRC-3 might alter the TIME to suppress breast cancer progression and inhibit oncogenic cellular pathways in cancers. Using a syngeneic mouse model of breast cancer, here, we found that SRC-3 inhibition by SI-2 treatment and SRC-3 knockdown generated tumor-suppressing TIME by recruiting cytotoxic immune cells changing through Cxcl9 elevation in breast tumors to aid in the suppression of breast tumor progression.

## Materials and methods

### Animal studies

C57BL/6J, C57BL/6J-SCID, B6 albino, BALB/cJ, and SCID mice were purchased from Jackson Laboratory. All animal studies were conducted with the approval of the Institutional Animal Care and Use Committee at Baylor College of Medicine. All mice were euthanized in a CO_2_ chamber. Additionally, any animals that showed a loss of mobility, 10% weight loss, or other symptoms related to tumor growth were euthanized. Tumors were not allowed to grow to > 20% of body weight or ulcerate. All animals were euthanized, and tumors were harvested at a volume of no more than 4,000 mm^3^.

### Animal number and power calculation

**Fig. 1:**
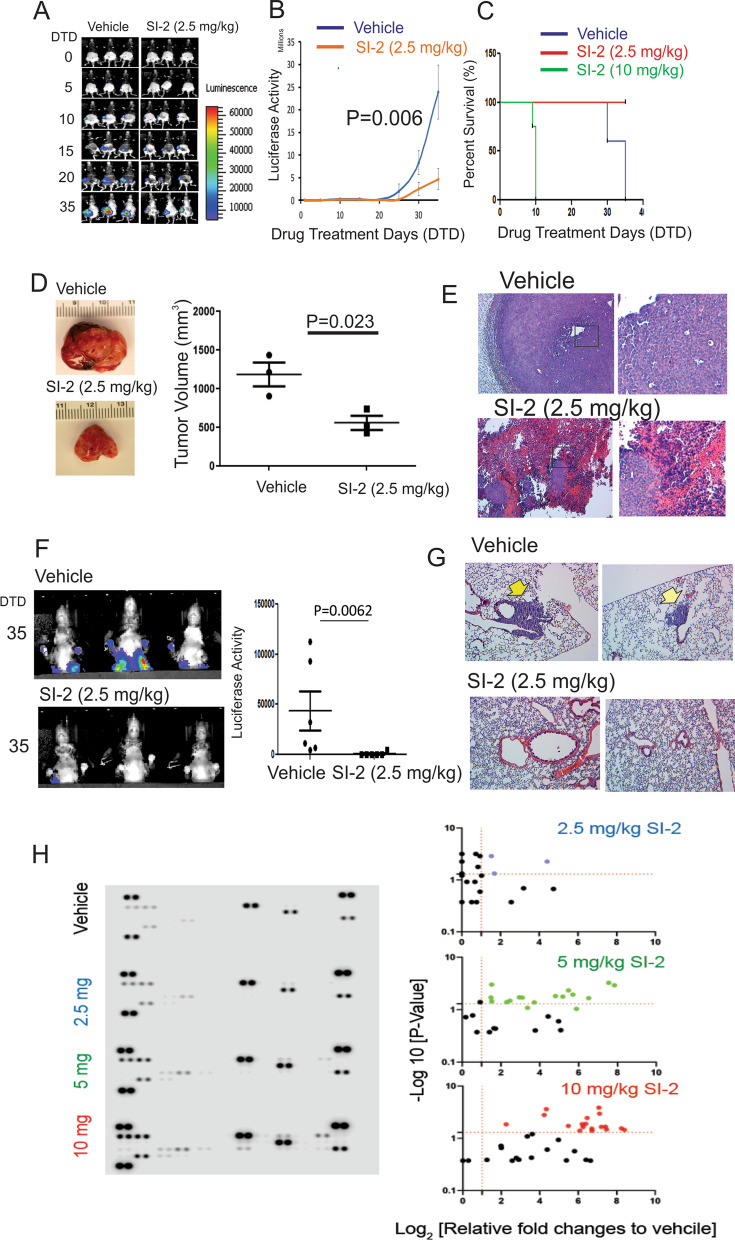
SI-2 suppresses the growth and lung metastasis of E0771 mammary gland tumors in C57BL/6J mice. **A** Reduction in luciferase activity in E0771 tumors from C57BL/6J mice by SI-2 (2.5 mg/kg) treatment compared with vehicle treatment. **B** Quantification of luciferase activity in the E0771 breast tumors shown in Panel A. **C** Kaplan–Meier plot of mice bearing E0771 tumors treated with SI-2 (2.5 and 10 mg/kg) or vehicle. If the luciferase activity in E0771 tumors in a mouse was greater than 20 × 10^6^ luciferase photons/second/cm^2^, the mouse was counted as deceased. The Kaplan–Meier plot was generated using GraphPad Prism (version 8.0). **D** Images and volumes of harvested E0771 tumors treated with SI-2 (2.5 mg/kg) or vehicle. Tumor volume was calculated as 0.5 × length × width × width. The analysis of the tumor volume in mice treated with SI-2 or vehicle is shown in the graph. **E** H&E staining of E0771 breast tumors treated with SI-2 (2.5 mg/kg) or vehicle. **F** Images and quantitative analysis of luciferase activity in E0771 cells in the lungs of mice treated with SI-2 (2.5 mg/kg) or vehicle, as shown in Panel A. **G** H&E staining of lungs harvested from E0771 tumor-bearing mice treated with SI-2 (2.5 mg/kg) or vehicle, as shown in Panel F. Lungs were serially sectioned at 7-µm intervals, and the slices with cancer masses were selected and then stained. Arrowheads indicate tumor masses in the lungs. **H** Blood cytokine profiles in female mice treated with vehicle or 2.5, 5, or 10 mg/kg SI-2 twice a day for 7 days. The relative fold change of each cytokine of blood from animals treated with SI-2 was calculated compared to the vehicle controls

In the luciferase activity analysis of E0771 tumor growth in Fig. 1A, the mean luciferase activity in mice treated with the vehicle was 23,923,333, and the standard deviation was 6,012,964. The mean luciferase activity in mice treated with SI-2 (2.5 mg/kg) was 4,672,000, and the standard deviation was 2,324,967 on the 35^th^ day after drug treatment. Therefore, the power calculation based on these data indicated that the animal size (n = 2/group) was sufficient to see a significant effect on E0771 breast cancer growth (*p* < 0.05).

Regarding the tumor size determination (Fig. 1D), the tumor volume in mice treated with the vehicle was 1,250 mm^3^ ± 155 and the tumor volume in mice treated with SI-2 (2.5 mg/kg) was 590 mm^3^ ± 57 on the 35th day after drug treatment. Therefore, the power calculation indicated that the optimum number of animals needed to attain a significance level of *p* < 0.05 with a statistical power of 95% was one.

### Cultures of the E0771, 4T1, and MDA-MB-468 breast cancer cell lines

E0771 (CRL-3461, ATCC) and 4T1 cells were grown in RPMI 1640 containing 10% (vol/vol) fetal bovine serum (FBS). MDA-MB-468 cells were cultured in DMEM (high glucose) containing 10% (vol/vol) FBS. The E0771, 4T1, and MDA-MB-468 cell lines were maintained in a humidified incubator with 5% (vol/vol) CO_2_ at 37 °C.

### Generation of luciferase-labeled E0771 and 4T1 cells

The firefly luciferase gene was cloned into the pSMPUW-Hygro Lentiviral Vector (Cell Biolabs, catalog number: VPK-214). Lentiviruses containing a luciferase expression cassette were produced in 293 TN cells (System Bioscience, catalog number: LV900A-1) by transient transfection of the pSMPUW-Hygro Lentiviral Vector carrying the luciferase gene and the ViraSafe™ Lentiviral Packaging System (Cell Biolabs, catalog number: VPK-206) with Lipofectamine 2000 (Thermo Fisher Scientific, catalog number: 11668030). The recombinant lentivirus titer was measured using Lenti-X™ GoStix™ Plus (ClonTech, catalog number: 631280). E0771 and 4T1 cells were transduced with lentiviral vectors carrying the luciferase expression cassette with TransDux MAX™ (System Bioscience, catalog number: LV860A-1). Luciferase-labeled E0771 and 4T1 cells were then selected in the presence of 300 µg/ml hygromycin. Luciferase gene expression in E0771 and 4T1 cells was validated using a luciferase activity assay kit (Promega, catalog number: E4550). Luciferase-labeled E0771 and 4T1 cells were maintained in RPMI 1640 medium supplemented with 10% FBS, penicillin/streptomycin, and 300 µg/ml hygromycin.

### Generation of SRC-3 knockdown (KD) E0771 and 4T1 cells

We purchased a pGIPZ vector containing an shRNA against the mouse SRC-3 gene (Thrmo Fisher, catalog number: V2LMN_24526). The pGIPZ vector containing a scrambled nontargeting (NT) shRNA (Thermo Fisher, catalog number: RHS4346) was used as the control. Lentivirus vectors expressing shSRC-3 and the NT shRNA were produced in 293 TN cells by transiently transfecting pGIPZ carrying shSRC-3 (or NT shRNA) and the ViraSafe™ lentiviral packaging system with Lipofectamine 2000. The recombinant lentivirus titer was measured using Lenti-X™ GoStix™ Plus. Luciferase-labeled E0771 and 4T1 cells were transduced with lentiviral vectors expressing shSRC-3 (or nontarget shRNA) with TransDux MAX™ and then selected in the presence of 1 µg/ml puromycin. The efficiency of SRC-3 KD in luciferase-labeled E0771 and 4T1 cells was determined by performing Western blotting with an SRC-3 antibody (rabbit, Cell Signaling Technology, catalog number: 2126; 1:1,000 dilution), and the results were compared to parental luciferase-labeled E0771 cells.

### Orthotopic injection of luciferase-labeled breast cancer cells into the mammary fat pads of syngeneic female recipient mice

A total of 1 × 10^5^ luciferase-labeled E0771 cells, SRC-3 KD luciferase-labeled E0771 cells, and luciferase-labeled E0771 cells expressing NT shRNA were resuspended in 50 µl of phosphate-buffered saline (PBS). They were then injected into one of the 4^th^ mammary fat pads in female C57BL/6J mice (8 weeks old), female B6 albino (8 weeks old), or female *C57BL/6J-SCID* mice (8 weeks old), as described in previous studies [17]. For 4T1 cells, 1 × 10^5^ luciferase-labeled 4T1 cells, SRC-3 KD luciferase-labeled 4T1 cells, and luciferase-labeled 4T1 cells expressing NT shRNA were injected into one of the 4th mammary fat pads in female BALB/cJ female mice (8 weeks old). Then, tumor growth was monitored by evaluating luciferase activity using an in vivo imaging system (IVIS). When the luciferase activity was 1000 luciferase photons/second/cm^2^ (~ 7 days after injection), the mice were randomized into four groups (*n* = 6 mice/group). Mice in the treatment group received an intraperitoneal injection of SI-2 (2.5, 5, or 10 mg/kg, twice a day for 35 days) or vehicle (phosphate-buffered saline containing 10% DMSO, 30% PEG300, and 1% Tween 20). Luciferase activity in luciferase-labeled E0771 and 4T1 breast tumors was evaluated twice a week for 5 weeks using IVIS.

### IVIS analysis of luciferase activity in mice

Mice were anesthetized with a 1.5% isoflurane/air mixture using an inhalation anesthesia system (VetEquip). D-Luciferin (Xenogen, catalog number: 122799) was injected intraperitoneally at a dosage of 40 mg/kg mouse body weight. Ten minutes after the D-luciferin injection, mice were imaged using the IVIS (Xenogen) under continuous exposure to 1% to 2% isoflurane. Imaging parameters were maintained for comparative analysis. Grayscale and pseudocolor images showing bioluminescence were superimposed and analyzed using Living Image software (Version 4.4, Xenogen). A region of interest (ROI) was manually selected over the relevant areas showing a luciferase signal. The ROI area was kept constant across the experiments, and the luciferase activity was recorded as total luciferase photon counts/second/cm^2^ within the ROI.

### SI-2 treatment to tumor-bearing mice

E0771 cells and 4T1 cells (1 × 10^6^ cells) were injected into the mammary fat pads of female mice (8 weeks old) to induce breast tumor formation. The tumor-injected female mice were randomly divided into two groups after detecting tumor growth using luciferase activity imaging (1000 luciferase photons/second/m2). Then, the mice in one group were treated with SI-2 (2.5 mg/kg, twice a day for 35 days), while mice in the control group were treated with vehicle alone.

### Kaplan–Meier survival analysis

Mice were considered deceased when the tumor had 20 × 10^6^ luciferase photons/second/cm^2^ because the volume of a tumor with this luciferase activity is approximately 20 mm in diameter, which is the maximum allowable tumor size according to our institution’s animal care guidelines. Kaplan–Meier survival analysis results were plotted using GraphPad Prizm (Version 8.0).

### Immunohistochemistry

Fresh mouse tumor tissues were fixed with 10% buffered formalin and then embedded in paraffin for subsequent sectioning. Tissue slices were deparaffinized by sequential soaking in xylene, ethanol, and water. Antigen retrieval was performed with antigen unmasking solution (Vector Laboratory, catalog number: H-3300). Endogenous peroxidase activity was blocked with a 3% (vol/vol) hydrogen peroxide solution. Sections were blocked with 2.5% (wt/vol) normal goat serum to reduce nonspecific antibody binding. Slices were incubated with primary antibodies, including anti-cleaved caspase 3 (rabbit, Cell Signaling Technology, catalog number: 9664, 1:300 dilution), anti-Ki-67 (rabbit, Cell Signaling Technology, catalog number: 9027; 1:1,000 dilution), anti-CD4 (rabbit, Abcam, catalog number: ab183685, 1:300 dilution), anti-CD8 (rabbit, Abcam, catalog number: ab217344, 1:300 dilution), anti-CD56 (rabbit, Novus Biologicals, catalog number: NBP2-38452, 1:300 dilution), anti-IFNG (rabbit, Novus Biologicals, catalog number: NBP2-66900, 1:300 dilution), anti-Cxcl9 (rabbit, Thermo Fisher Scientific, catalog number: PA5-81371, 1:100 dilution), and anti-Foxp3 (rabbit, Invitrogen, catalog number: PA1-16876, 1:300 dilution) antibodies, at 4 °C overnight. Slices were stained with HRP polymer-conjugated secondary antibodies (Vector Laboratories, catalog number: 7401 for rabbit antibodies). Slices were reacted with a freshly prepared DAB solution (Dako, catalog number: K3468). After staining, nuclear counterstaining was performed with Mayer's hematoxylin.

### Dual immunofluorescence staining

Tissue slices were deparaffinized by sequential soaking in xylene, ethanol, and water. Antigen retrieval was performed with antigen unmasking solution (Vector Laboratory, catalog number: H-3300). Slides were blocked with 2.5% (wt/vol) normal goat serum to reduce nonspecific antibody binding. Slides were incubated with anti-CD4 (rabbit, Abcam, catalog number: ab183685, 1:100 dilution) and anti-FOXP3 (rat, LSBio, catalog number: LS-C344878, 1:100 dilution) antibodies at 4 °C overnight. Afterward, the slides were washed with TBST [20 mM Tris (pH 7.5), 150 mM NaCl and 0.1% (w/v) Tween 20]. They were then incubated with goat anti-rabbit Alexa Fluor 488 (Thermo Fisher, catalog number: A11008, 1:500 dilution) and goat anti-rat Alexa Fluor 594 (Thermo Fisher, catalog number: A11007, 1:500 dilution) for 1 h at room temperature. Afterward, slides were washed with TBST and mounted with Antifade Mounting Medium containing DAPI (Vector, catalog number: H-2000).

### Western blot analyses

Primary antibodies against the following proteins were used: ERα (Santa Cruz Biotechnologies, catalog number: SC-71064, 1:1000 dilution), ERβ (Santa Cruz Biotechnologies, catalog number: SC-373853, 1:1000 dilution), PR (Santa Cruz Biotechnologies, catalog number: SC-7208, 1:1000 dilution), HER-2 (Cell Signaling, catalog number: 2165, 1:1000 dilution), SRC-3 (Cell Signaling, catalog number: 2126, 1:1000 dilution), and β-Actin (Santa Cruz Biotechnologies, catalog number: SC-47778, 1:1000 dilution). Membranes containing proteins were incubated with a goat anti-rabbit IgG-HRP antibody (Sigma, catalog number: A0545, 1:5000 dilution), and the signals were visualized using an ECL plus kit (Thermo Scientific, catalog number: 11517271).

### Mouse cytokine antibody array assay

E0771 breast tumors were isolated from mice on the 21st day after SI-2 or vehicle treatment. Afterward, cell lysates were generated from breast tumors treated with SI-2 and vehicle using lysis buffer [20 mM Tris HCl (pH 7.5), 150 mM NaCl, 10% glycerol, 1% Nonidet P-40 (NP-40), and 2 mM EDTA]. Additionally, SRC-3 KD E0771 breast tumors and their control E0771 breast tumors were harvested on the 14th day after tumor cell injection. Cell lysates were generated from SRC-3 KD E0771 breast tumors and their control tumors with lysis buffer. According to the manufacturer's protocols, cytokine levels in cancers were determined with a Proteome Profiler Mouse Cytokine Antibody Array Kit (R&D Systems, catalog number: ARY006).

### SI-2 toxicity determination

C57BL/6J female mice (8 weeks old, *n* = 5/group) were intraperitoneally treated with vehicle or 2.5, 5, or 10 mg/kg SI-2 (twice a day) for one week. Afterward, blood was extracted from the mice and according to the manufacturer's protocols, cytokine levels in blood were determined using the Proteome Profiler Mouse Cytokine Antibody Array Kit (R&D Systems, catalog number: ARY006).

### 3-(4,5-Dimethylthiazol-2-yl)-5-(3-carboxymethoxyphenyl)-2-(4-sulfophenyl)-2H-tetrazolium) (MTS) cell growth assay

E0771 cells were inoculated into the wells of 96-well plates (1 × 10^4^ cells/well). The next day, each cell line was treated with serially diluted IL-1RA (0–200 ng/ml) and TIMP-1 (0–200 ng/ml). After 3 days, 10 μL of MTS reagent in Premix WST-1 Cell Proliferation Assay system (Takara, catalog number: MK400) was added to each well. MTS-treated plates were incubated for 2 h. Then, the optical density of the color in each well was measured at 450 nm using a microtiter plate reader.

### Determination of the IC_50_ values of SI-2 in different breast cancer cells

E0771, 4T1, and MDA-MB-486 cells were plated into 96-well plates (1 × 10^4^ cells/well). The next day, each cell line was treated with serial dilutions of SI-2. On the 3rd day after SI-2 treatment, the proliferation of breast cancer cells was determined using the MTS assay.

### RNA analysis of Cxcl9, Il-1ra, and Il-16 in SRC-3 KD and KD control E0771 cells

SRC-3 KD and KD control E0771 cells were grown in RPMI 1640 containing 10% (vol/vol) FBS and 1 μg/ml puromycin. Total RNA was isolated from cells using a RNeasy Kit (Qiagen, catalog number: 74004). cDNA was generated from 1 μg of total RNA from cells using a high-capacity reverse transcription kit (Applied Biosystems, catalog number: 4368814). The expression levels of target genes were determined by quantitative PCR with SsoAdvanced Universal SYBR Green Supermix (BIO-RAD, catalog number: 1725271) and target gene primers as follows: Cxcl9 (5’-ACTCCAACACAGTGACTCAATAG -3' and 5’-CGTTCTTCAGTGTAGCAATGATTT-3'), Il-1ra (5’-CAAGCCTTCAGAATCTGGGATA-3', and 5’-CTCAGAGCGGATGAAGGTAAAG-3'), and Il-16 (5’-GCGTCAGTCATCTCCAACATAG-3' and 5’-CCAACACCTGCCTCTTTCTT-3').

### Statistical analyses

Data are presented as the mean ± SEM values. Statistical comparisons between two groups were made with one-way ANOVA, followed by Student's t test or the Mann–Whitney U test. Sidak's multiple comparison test was used for comparisons among more than two groups. A *P* value < 0.05 was considered significant. GraphPad Prism (version 8.0) software was used for statistical analyses.

## Results

### SI-2 treatment suppressed the growth and lung metastasis of E0771 breast tumors in immune-intact syngeneic C57BL/6J mice

To determine the role of SRC-3 in the modulation of TIME, we employed an E0771 murine mammary cancer cell line that was initially isolated from a spontaneous tumor in a C57BL/6 mouse [18]. Luciferase-labeled E0771 cells were generated by a lentivirus carrying a luciferase expression cassette to noninvasively evaluate tumor growth in mice. Since luciferase expression does not alter metabolism in cancer cells [19], the luciferase activity measured from luciferase-expressing E0771 breast tumors facilitated the practical quantification of tumor development in recipient mice. As shown in our previous study, continuous treatment of mice with SI-2 (2 mg/kg, twice a day, for 5 weeks) suppressed MDA-MB-468 breast cancer progression in severe combined immunodeficiency (SCID) mice without inducing toxicities in internal organs [11]. When cancer growth was evaluated by detecting luciferase activity (~ 1000 luciferase photons/second/cm^2^), E0771 breast cancer-bearing C57BL/6J female mice were treated with SI-2 (2.5 mg/kg, twice a day for 35 days) and drug vehicle as the control based on our previously established xenograft mouse model[11]. Comparative analyses of the luciferase activity images revealed that the E0771 cells robustly formed breast tumors in syngeneic C57BL/6J mice-administered vehicle treatment (Fig. 1A, B). However, SI-2 treatment significantly reduced imaging-based luciferase activity in E0771 breast tumors compared to vehicle-treated breast tumors (Fig. 1A, B). Therefore, SI-2 treatment effectively suppressed the growth of E0771 breast tumors in immune-intact syngeneic C57BL/6J mice. All E0771 breast cancer-bearing mice treated with vehicle were euthanized on 35 days after cancer cell injection because tumors were reached the maximum size of tumors (> 20 mm in diameter) (Fig. 1C, D). However, compared to vehicle-treated mice, SI-2 (2.5 mg/kg)-treated mice had a small tumor size but did not have the maximum tumor size (< 10 mm in diameter) on the 35th day after the injection of cancer cells (Fig. 1C, D). Collectively, this result substantiates that compared to vehicle treatment, SI-2 treatment effectively suppressed the growth of E0771 breast tumors in immune-intact C57BL/6J mice.

In addition to evaluating luciferase activity in images, we harvested tumors from animals treated with SI-2 and vehicle on the 35th day after tumor cell injection and determined tumor volumes. We then conducted a histological analysis of the harvested tissue. Consistent with the luciferase activity in the tumors, the analysis of the tumor volume revealed that compared to vehicle treatment, SI-2 treatment significantly reduced the tumor volume (2.1-fold, *p* = 0.023) (Fig. 1D). Hematoxylin and eosin (H&E) staining revealed SI-2 treatment-induced hypocellularity in cancers compared with vehicle treatment because an organized structure was not detected in the tumor masses treated with SI-2 (Fig. 1E). SI-2 effectively suppressed breast cancer progression in immune-intact mice.

E0771 cells from primary breast tumors metastasize to the lungs of C57BL/6J mice [20]. Thus, we determined whether SI-2 inhibited lung metastasis of primary E0771 breast tumors that developed in C57BL/6J mice. An analysis of luciferase activity images from our cancer-bearing mice revealed that luciferase activity from E0771 cells was detected in the lungs on the 35th day after vehicle treatment (Fig. 1F). However, unlike the vehicle treatment, luciferase activity in E0771 cells was not detected in the lungs of E0771 tumor-bearing mice on the 35th day after SI-2 treatment (Fig. 1F). We isolated lungs from cancer-bearing mice treated with SI-2 or vehicle and performed H&E staining of the tissue to validate the difference in lung metastasis identified by imaging luciferase activity. H&E staining analysis with the serial section of the lung revealed that tumor masses were detected in the lungs of cancer-bearing mice treated with vehicle, but cancer masses were not detected in the lungs of mice treated with SI-2 (Fig. 1G). In addition to suppressing the growth of primary breast tumors, SI-2 also effectively inhibited lung metastasis of E0771 breast tumors in C57BL/6J mice.

In contrast to the low-dose-treated animals (2.5 mg/kg of SI-2), mice were euthanized 10 days after administration of the high dose of SI-2 (10 mg/kg) because mice were notably sick (Fig. 1C). Compared to administration with 10 mg/kg, however, administration of 5 mg/kg SI-2 did not lead to sickness in these tumor-bearing mice. Moreover, the cancer-suppressive activity of 5 mg/kg SI-2 was less than that of 2.5 mg/kg SI-2 (Additional file 1: Fig. S1A). To understand this observation, the cytotoxicity of SI-2 was determined in C57BL/6J female mice (8 weeks old) treated with vehicle or 2.5, 5, or 10 mg/kg SI-2 twice a day for one week. Comparative analysis of the cytokine profile of blood in these animals revealed that 10 mg/kg SI-2 treatment significantly increased the levels of various cytokines in blood compared to the vehicle-treated animals (Fig. 1H and Additional file 2: Fig. S2). Therefore, the cytotoxicity of 10 mg/kg SI-2 leads to sickness in mice. SI-2 (5 mg/kg) also elevated the levels of cytokines in the blood compared to the vehicle, even though the fold changes in cytokines induced by 5 mg/kg SI-2 were less than those induced by 10 mg/kg SI-2 (Fig. 1H and Additional file 2: Fig. S2). However, 2.5 mg/kg SI-2 increased the levels of only three cytokines in the blood compared to the vehicle (Fig. 1H and Additional file 2: Fig. S2). 5 mg/kg of SI-2 slightly increased CD4 + T cells, but did not change the level of CD8 + T cells in breast tumors compared to 2.5 mg/kg of SI-2 (Additional file 1:Fig. S1B, C). Interestingly, 5 mg/kg of SI-2 significantly reduced CD56 + NK cell levels in E0771 breast tumors in C57BL/6J mice compared to 2.5 mg/kg of SI-2 (Additional file 1:Fig. S1D). Therefore, the high levels of blood cytokines likely reduce antitumor activity upon administration with 5 mg/kg of SI-2 compared to administration of 2.5 mg/kg of SI-2 by altering the tumor immune microenvironment.

### SI-2 treatment reduced the proliferation and increased the apoptosis of E0771 breast cancer cells

**Fig. 2:**
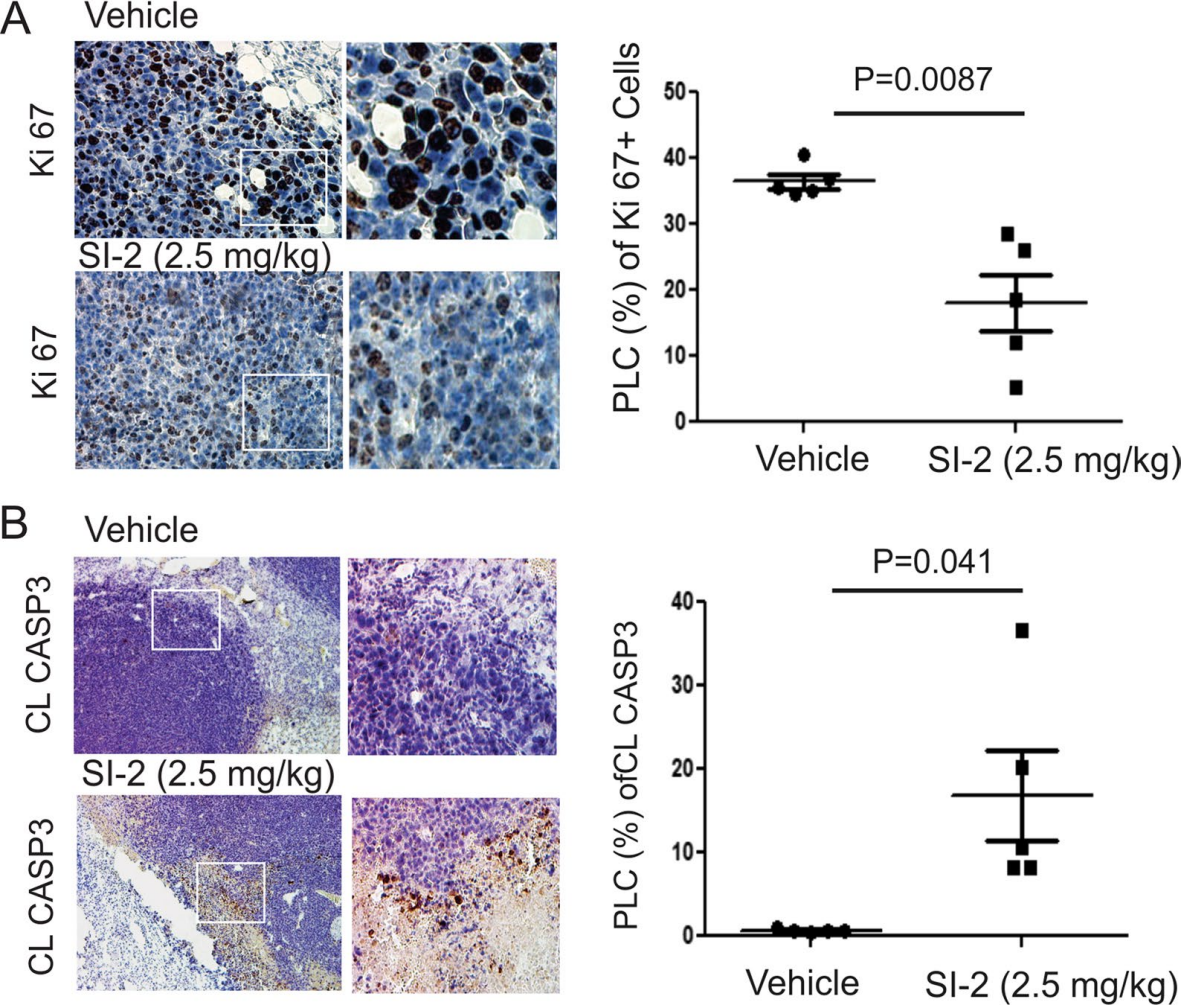
SI-2 treatment inhibits the proliferation and activates apoptosis of E0771 breast tumor cells. **A** Expression levels of Ki67 in E0771 tumors from C57BL/6J mice treated with SI-2 (2.5 mg/kg) or vehicle were determined using IHC with an antibody against Ki67. The quantification of the percentage of labeled cells (PLC) positive for Ki67 in E0771 breast tumors is shown in the graph. **B** Expression levels of cleaved caspase 3 (CL CASP3) in E0771 tumors treated with SI-2 (2.5 mg/kg) or vehicle were determined using IHC with an antibody against cleaved caspase 3. The quantification of the PLC positive for CL CASP3 in E0771 tumors is shown in the graph

According to a previous study, SI-2 effectively suppressed MDA-MB-468 breast cancer progression in SCID mice by inhibiting tumor cell proliferation [11]. In immune-intact C57BL/6J mice, SI-2 treatment also significantly reduced the percentage of proliferating Ki67-expressing cells compared to their vehicle-treated counterparts (Fig. 2A). Furthermore, compared with vehicle treatment, SI-2 treatment increased apoptosis in MDA-MB-468 cells in immunodeficient mice to inhibit tumor growth [11]. Therefore, we wanted to investigate whether SI-2 also increased apoptosis in aggressive E0771 breast tumors in C57BL/6J mice. Immunohistochemistry (IHC) with an anti-cleaved caspase 3 antibody revealed that SI-2 treatment significantly increased the percentage of cleaved caspase 3-expressing cells compared to vehicle treatment (Fig. 2B). Thus, SI-2 treatment promoted apoptosis in E0771 breast tumors to suppress breast tumor growth in immune-intact mice.

### SI-2 treatment generated antitumor TIME in E0771 breast tumors

**Fig. 3:**
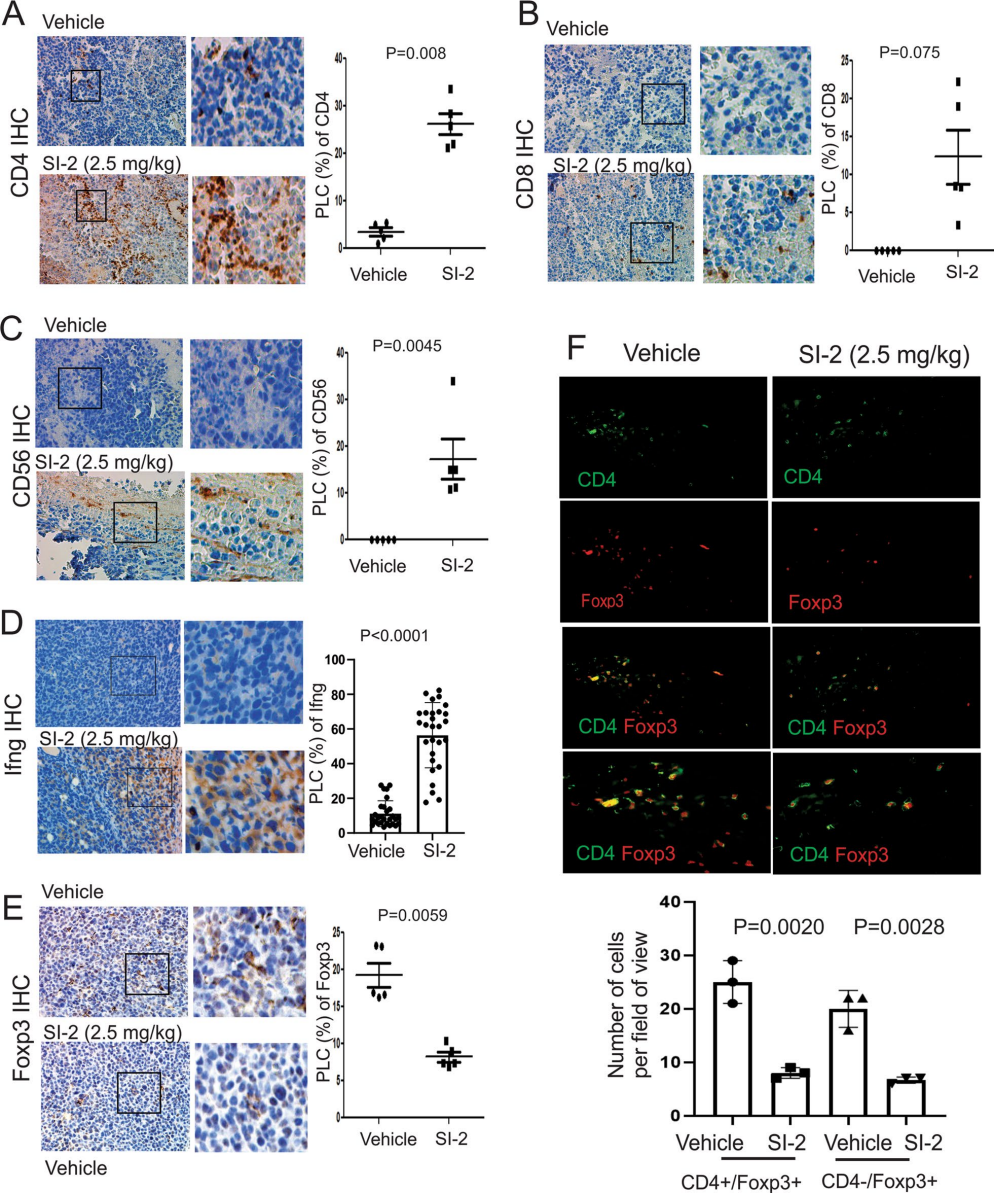
SI-2 treatment alters the tumor-infiltrating immune cell repertoire in E0771 tumors. **A** Numbers of CD4 + T cells in E0771 tumors in C57BL/6J mice treated with SI-2 (2.5 mg/kg) or vehicle, as determined using IHC with an antibody against CD4. Quantification of the percentage of labeled cells (PLC) with CD4 in E0771 tumors is shown in the graph. **B** Numbers of CD8 + T cells in E0771 breast tumors treated with SI-2 (2.5 mg/kg) or vehicle, as determined using IHC with an antibody against CD8. Quantification of the PLC for CD8 in E0771 tumors is shown in the graph. **C** Numbers of CD56 + cells in E0771 tumors treated with SI-2 (2.5 mg/kg) or vehicle, as determined using IHC with an antibody against CD56. The PLC for CD56 is shown in the graph. **D** Numbers of Ifng-expressing cells in E0771 tumors treated with SI-2 (2.5 mg/kg) or vehicle examined by IHC with Ifng antibody. The PLC for Ifng is shown in the graph. **E** Numbers of Foxp3 + T cells in E0771 tumors treated with SI-2 (2.5 mg/kg) or vehicle, as determined using IHC with an antibody against Foxp3. The PLC for Foxp3 in E0771 tumors is shown in the graph. **F** Dual immunofluorescence staining to determine CD4 + (green), Foxp3 + (red), and CD4 + /Foxp3 + or CD4-/Foxp3 + cells in E0771 breast tumors from mice treated with vehicle and SI-2 (2.5 mg/kg) for 35 days. The numbers of CD4 + /Foxp3 + and CD4-/Foxp3 + cells in E0771 breast tumors treated with vehicle and SI-2 (2.5 mg/kg) in Panel F are quantified

The differential TIME suppresses or stimulates breast cancer progression [21]. Thus, we wanted to determine whether SI-2 treatment altered the infiltrating immune cell repertoire in E0771 breast tumors to suppress their growth. Immunohistochemical staining revealed that SI-2 treatment increased the numbers of infiltrating CD4+, CD8+, and CD56+ cytotoxic immune cells in E0771 tumors compared to vehicle treatment (Fig. 3A–C). Cytotoxic CD4^+^ T cells directly or indirectly eliminate MHC class II-positive tumor cells [22]. CD8 + cytotoxic T cells also suppress MHC class I-positive tumor cells [23]. Therefore, SI-2 treatment suppresses E0771 breast tumor progression in immune-intact mice by recruiting CD4+ and CD8+ T cells into tumors. Hematopoietic expression of CD56 appears to be restricted to activated immune cells, such as natural killer (NK) cells, which exhibit cytotoxic activity that suppresses cancer progression [24, 25]. Cytotoxic immune cells produce interferon-gamma (Ifng) that enhances their cytotoxicity [26]. To define whether the recruited CD4+ and CD8+ T cells in breast tumors function as cytotoxic immune cells, Ifng levels were determined. IHC revealed that SI-2 treatment elevated the levels of Ifng in tumors compared to vehicle treatment (Fig. 3D). Therefore, SI-2 promoted the establishment of an antitumor immune environment at least in part by recruiting cytotoxic CD4+, CD8+ T cells, and CD56+ cells into the tumor tissue.

In contrast to its effects on cytotoxic immune cells, SI-2 treatment significantly reduced the number of Foxp3+ cells in E0771 breast tumors in C57BL/6J mice compared to vehicle-treated mice (Fig. 3E). Foxp3 is the crucial transcription factor that establishes regulatory T (Treg) cell identity and drives their immunosuppressive activity [27]. In addition to Treg cells, previous studies revealed that Foxp3 is also expressed in various cancers and promotes tumor progression by activating cancer-specific cellular pathways, such as the Wnt/β-catenin signaling pathway and EMT [28, 29]. Thus, we need to validate whether the reduction in Foxp3 expression correlated with a lower number of Treg cells. We conducted dual immunofluorescence staining to address this question. Both CD4 + /Foxp3 + and CD4-/Foxp3 + cells were detected in control E0771 breast tumors (Fig. 3F). CD4 + /Foxp3 + cells are Tregs, and CD4-/Foxp3 + cells might be E0771 breast cancer cells expressing Foxp3. Compared to the control, SI-2 treatment significantly reduced the numbers of both CD4 + /Foxp3 + and CD4-/Foxp3 + cells in E0771 breast tumors. Therefore, SI-2 treatment increased the ratio of cytotoxic lymphocytes to Tregs in E0771 tumors by reducing the Treg cell population, likely leading to enhanced antitumor immunity capable of limiting tumor progression.

### SI-2 treatment also suppressed 4T1 breast tumor progression in syngeneic immune-intact BALB/cJ female mice

**Fig. 4:**
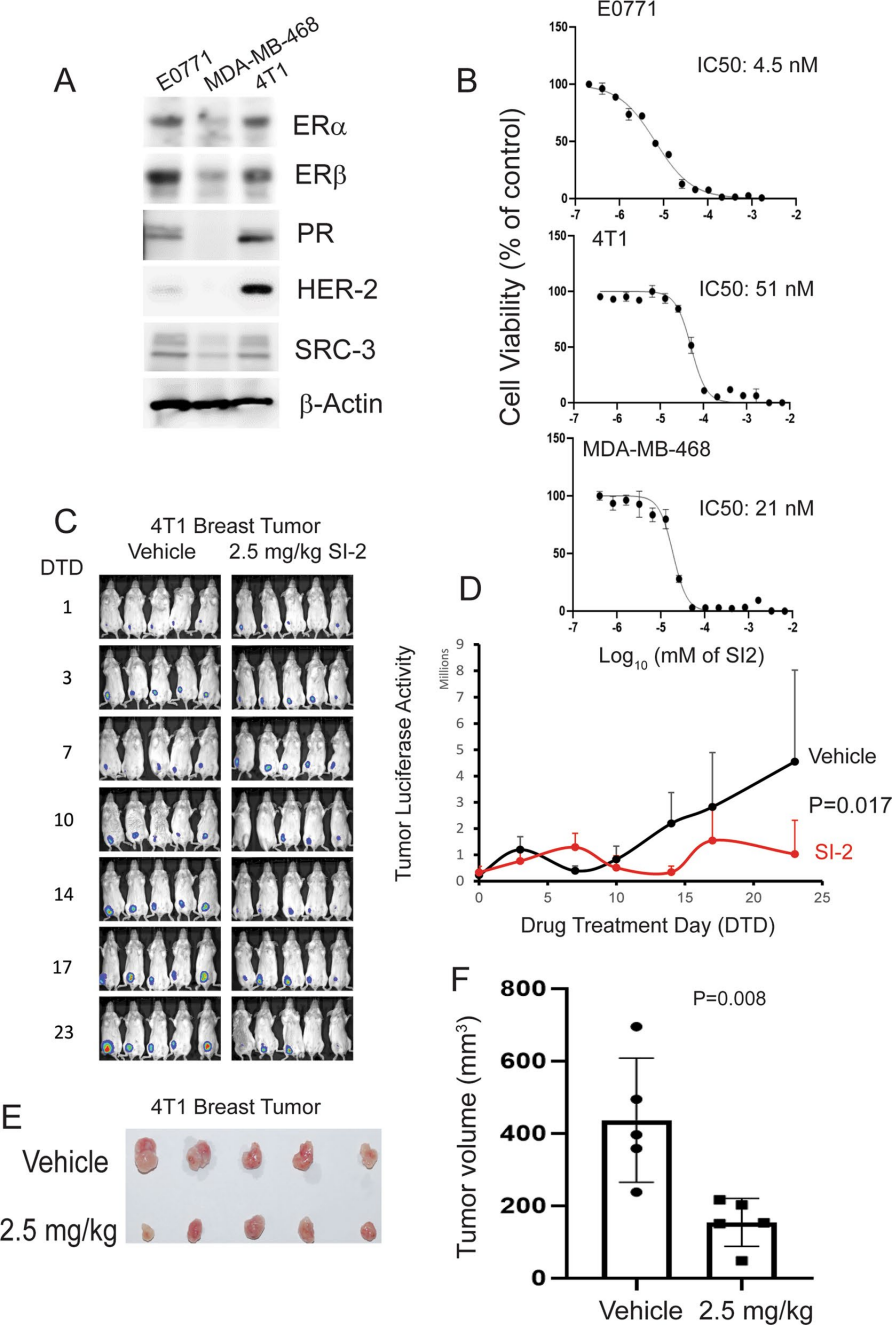
SI-2 suppressed 4T1 breast cancer progression in BALB/cJ mice. **A** Western blot analysis of ERα, ERβ, PR, HER2, SRC-3, and β-Actin levels in E0771, 4T1, and MDA-MB-468 cells. **B** Determination of the IC_50_ values of SI-2 against E0771, 4T1, and MDA-MB-468 cells. **C** Luciferase activity imaging analysis of the growth of luciferase-labeled 4T1 breast tumors in BALB/cJ mice treated with vehicle and SI-2 (2.5 mg/kg). **D** Quantification of the luciferase activity of 4T1 breast tumors in Panel C. **E** 4T1 breast tumors harvested from tumor-bearing BALB/cJ mice treated with vehicle and SI-2 (2.5 mg/kg) on the 23^rd^ day after drug treatment. **F** Quantification of the 4T1 tumor volume in Panel E. Tumor volume was calculated as 0.5 × length × width × width

We employed the 4T1 tumor-bearing syngeneic BALB/cJ mouse model to determine whether SI-2 could suppress the progression of another breast tumor line in a syngeneic mouse model of breast cancer [30]. First, we determined the expression levels of nuclear receptors, HER2, and SRC-3 in E0771 cells, 4T1 cells, and MDA-MB-468 cells. E0771 and 4T1 cells expressed estrogen receptor (ER)α, ERβ, and progesterone receptor (PR) at higher levels than MDA-MB-468 cells (Fig. 4A). Additionally, the SRC-3 expression levels in E0771 cells were similar to those in 4T1 cells, but HER-2 expression in E0771 cells was lower than that in 4T1 cells (Fig. 4A). Accordingly, the molecular profile of mouse E0771 and 4T1 cells was distinct from human MDA-MB-468 cell lines. Afterward, we determined the half-maximal inhibitory concentrations (IC_50_) of SI-2 for E0771, 4T1, and MDA-MB-468 cells. SI-2 suppressed the growth of all breast cancer cells (Fig. 4B). The growth of E0771 cells was suppressed by a low concentration of SI-2 (IC_50_: 4.5 nM) compared to 4T1 (IC_50_: 51 nM) and MDA-MB-468 cells (IC_50_: 21 nM) (Fig. 4B).

Luciferase-labeled 4T1 cancer cells were orthotopically injected into the mammary fat pad of immune-intact BALB/cJ female mice to determine whether SI-2 treatment also suppressed 4T1 breast tumor progression in syngeneic immune-intact BALB/cJ female mice in vivo. Animals were then intraperitoneally injected with 2.5 mg/kg SI-2 (twice a day) and the vehicle. SI-2 treatment significantly reduced the luciferase activity of 4T1 breast tumors in BALB/cJ mice compared to vehicle treatment (Fig. 4C, D). After the final luciferase imaging analysis, we harvested 4T1 breast tumors and determined their volumes. Consistent with the luciferase activity imaging analysis, SI-2 treatment significantly reduced 4T1 breast tumor volume compared to the vehicle (Fig. 4E, F). In addition to E0771 cells, SI-2 effectively suppressed 4T1 breast cancer progression in immune-intact female mice.

**Fig. 5:**
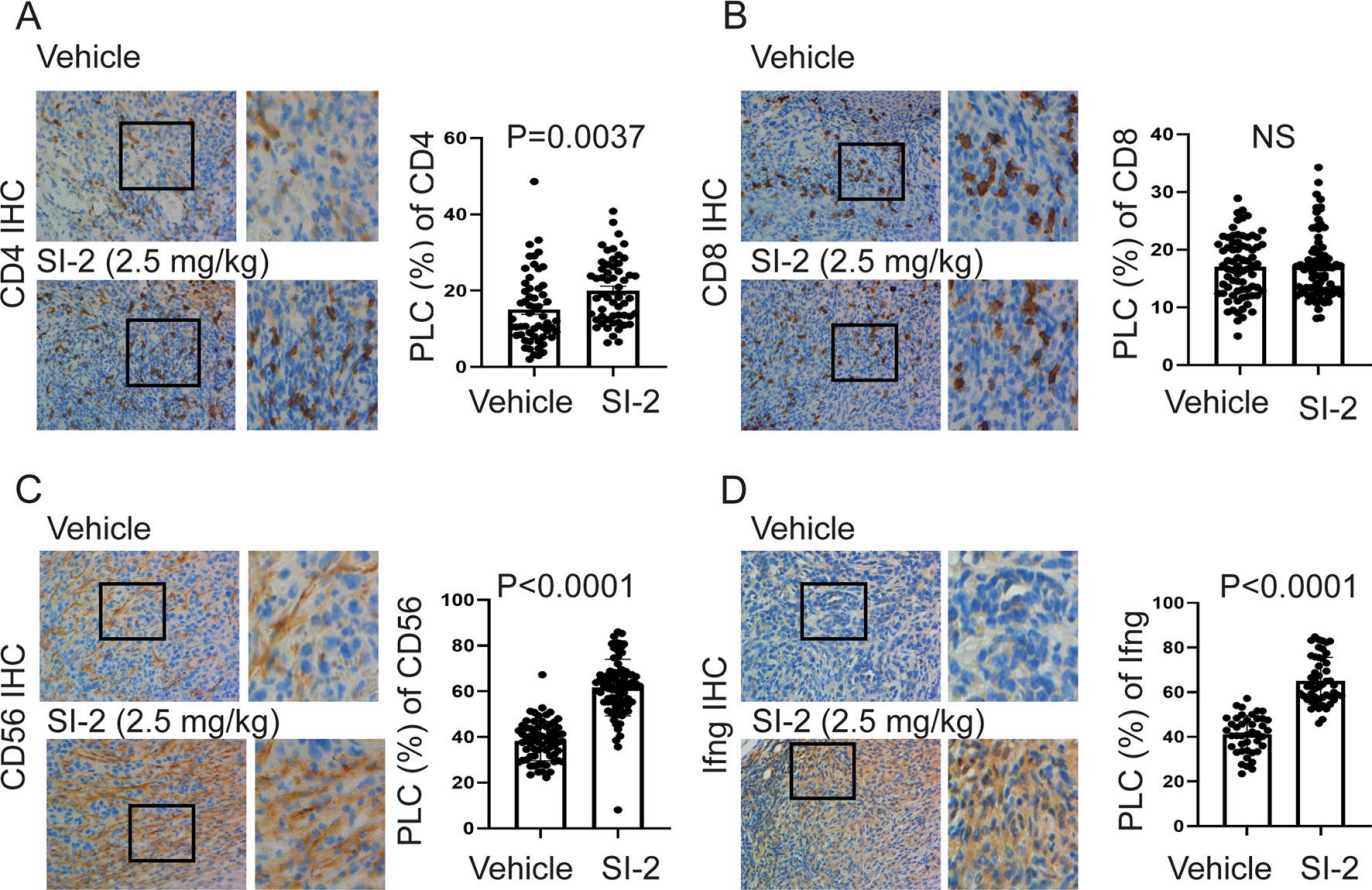
SI-2 treatment alters the tumor-infiltrating immune cell repertoire in 4T1 breast tumors. **A**–**D** Quantification of CD4 + T cells (**A**), CD8 + T cells (**B**), CD56 + NK cells (**C**) and Ifng (**D**) in 4T1 breast tumors in BALB/c mice treated with SI-2 (2.5 mg/kg) or vehicle, as determined by IHC with an antibody against their respective target protein. The quantification of the percentage of labeled cells (PLC) with CD4, CD8, CD56, and Ifng in 4T1 breast tumors is shown in the graph

Tumor-infiltrating immune cells in 4T1 breast tumors were also assessed to determine whether SI-2 treatment changes the TIME. Treatment with 2.5 mg/kg SI-2 elevated the levels of CD4 + and NK cells in 4T1 breast tumors compared to the vehicle controls (Fig. 5A, C). However, CD8 + levels were not changed in 4T1 breast tumors by SI-2 treatment compared to vehicle controls (Fig. 5B). SI-2 treatment significantly elevated Ifng levels in 4T1 breast tumors compared with vehicle controls (Fig. 5D). Collectively, SI-2 treatment also recruited cytotoxic immune cells (CD4 + T cells and NK cells) and then generated a tumor-suppressing TIME to suppress 4T1 breast tumor progression in immune-intact female mice.

### SRC-3 is the critical immune modulator in breast tumor progression

**Fig. 6:**
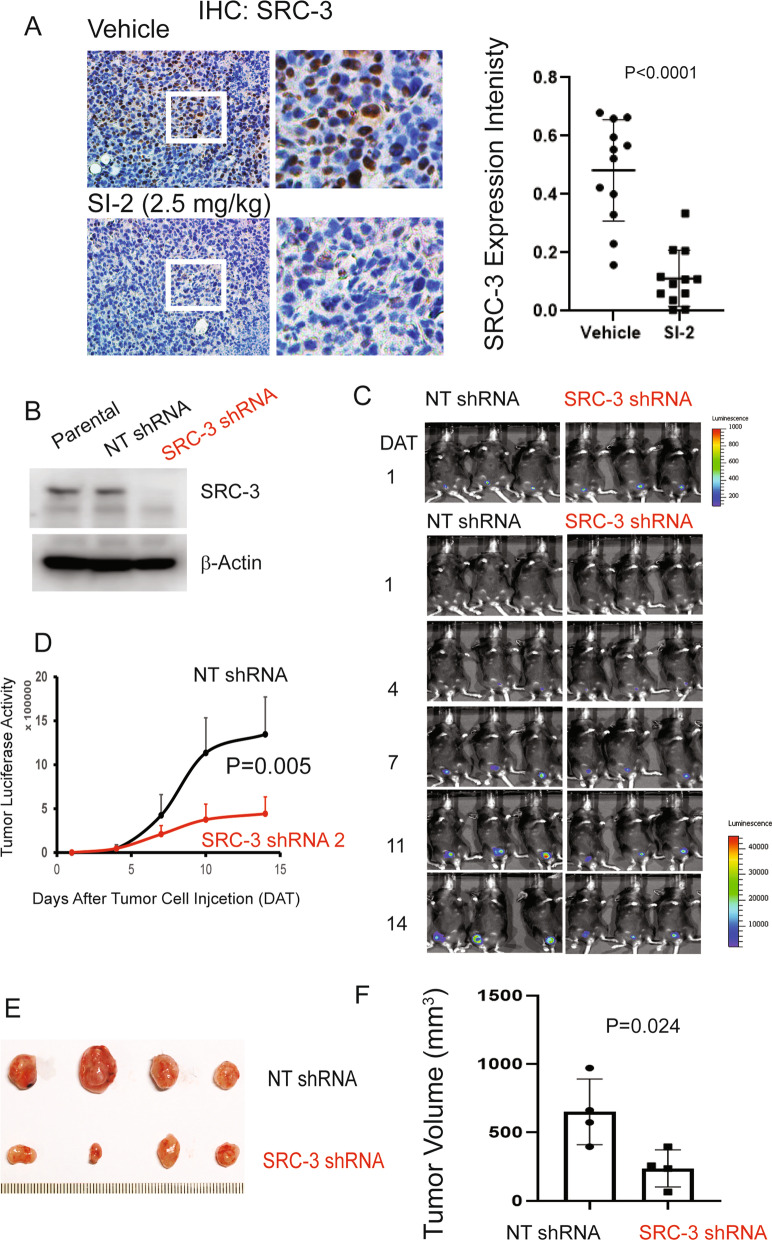
SRC-3 plays a critical role in E0771 breast cancer progression in C57BL/6J mice. **A** IHC analysis of SRC-3 levels in E0771 breast cancer cells treated with SI-2 (2.5 mg/kg) or vehicle after the 35^th^ day of drug treatment. **B** Western blot analysis of the SRC-3 levels in paternal E0771 cells and E0771 cells expressing a nontargeting shRNA and shRNA against mouse SRC-3. **C** Luciferase activity imaging analysis of the growth of luciferase-labeled SRC-3 KD E0771 breast tumors and control luciferase-labeled E0771 breast tumors expressing the nontargeting shRNA in C57BL/6J mice. **D** Quantification of the luciferase activity of luciferase-labeled SRC-3 KD E0771 and KD control E0771 breast tumors in Panel C. **E** SRC-3 KD and control breast tumors harvested from tumor-bearing C57BL/6J mice on the 14^th^ day after the injection of cancer cells. **F** Quantification of the volume of SRC-3 KD and control breast tumors in Panel E. Tumor volume was calculated as 0.5 × length × width × width

Our previous study revealed that SI-2 treatment reduced SRC-3 levels in E0771 cells in vitro [31]. Thus, we examined SRC-3 levels in E0771 breast tumors in C57BL/6J mice treated with SI-2 compared with vehicle. SI-2 treatment significantly reduced SRC-3 levels in E0771 breast tumors compared to the vehicle (Fig. 6A).

Next, we determined whether SRC-3 plays an essential role in E0771 breast cancer progression in immune-intact mice. We generated stable SRC-3 knockdown (KD) luciferase-labeled E0771 cells using a lentivirus expressing an shRNA against mouse SRC-3 and then determined the effect of the loss of SRC-3 function on luciferase-labeled E0771 breast tumor progression. The shRNA against SRC-3 significantly reduced SRC-3 levels in E0771 cells compared to parental E0771 cells and E0771 cells expressing a nontargeting (NT) shRNA (Fig. 6B). Afterward, SRC-3 KD luciferase-labeled E0771 cells were orthotopically injected into the mammary fat pads of recipient C57BL/6J female mice. As a control, luciferase-labeled E0771 cells expressing the NT shRNA were injected into the mammary fat pads of recipient C57BL/6J female mice. The luciferase activity of SRC-3 KD E0771 breast tumors was significantly lower than that of control E0771 breast tumors (Fig. 6C, D). After the final luciferase activity analysis, we harvested SRC-3 KD and control E0771 breast tumors and determined their volumes. The SRC-3 KD E0771 breast tumor volume was significantly lower than that of control E0771 breast tumors (Fig. 6E, F). SRC-3 played a critical role in E0771 breast cancer progression in immune-intact mice.

**Fig. 7:**
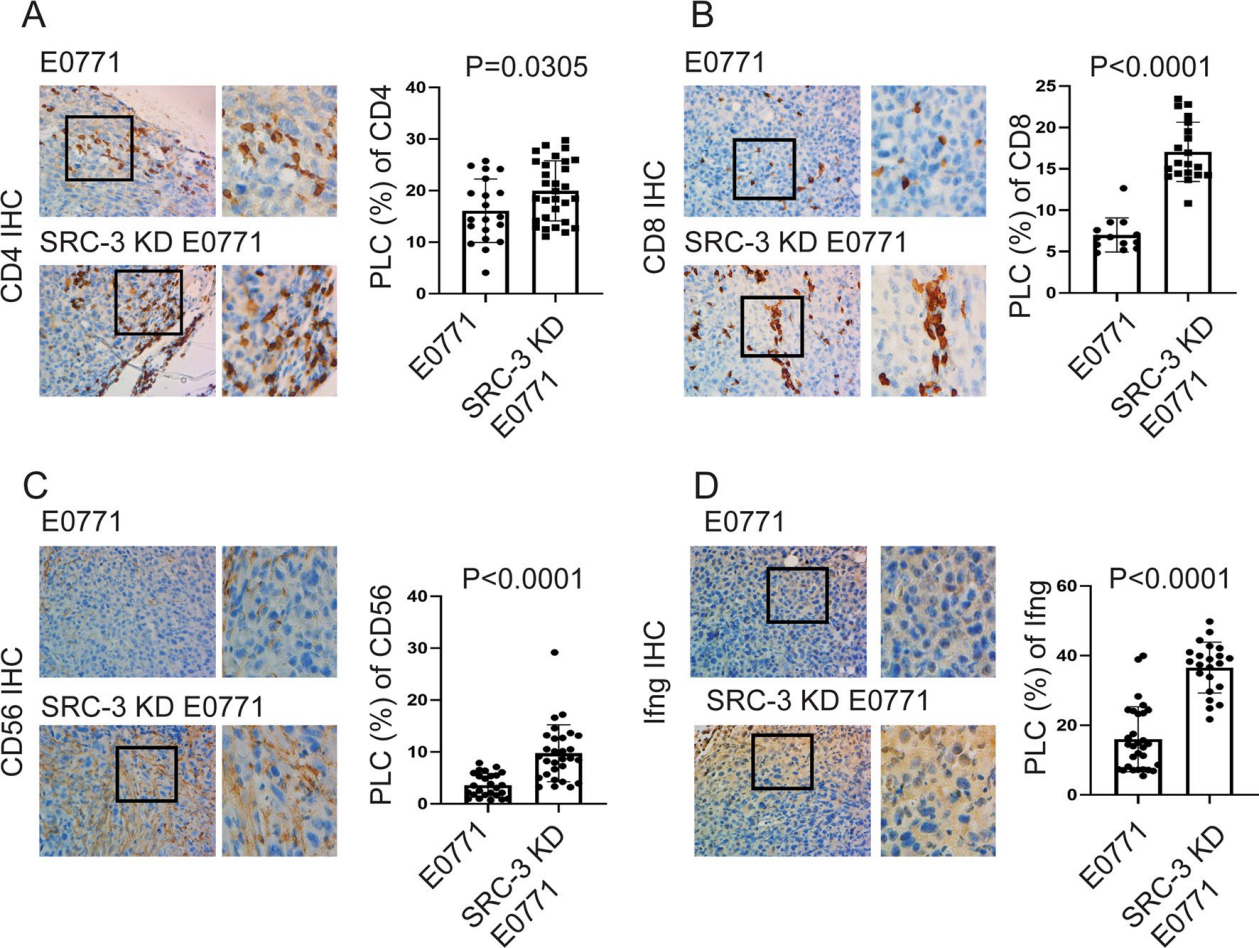
SRC-3 KD changes the tumor-infiltrating immune cell repertoire in E0771 breast tumors. **A-D** Quantification of CD4 + T cells (**A**), CD8 + T cells (**B**), CD56 + NK cells (**C**), and Ifng (**D**) in SRC-3 KD E0771 versus control KD breast tumors in C57BL/6J mice, as determined by IHC with antibodies against their respective target proteins. Quantifications of the percentages of labeled cells (PLC) with CD4, CD8, CD56, and Ifng in SRC-3 KD E0771 versus control KD breast tumors are shown in the graph

Next, we determined the composition of tumor-infiltrating immune cells in SRC-3 KD E0771 breast tumors. IHC analysis revealed that the levels of CD4 + T cells, CD8 + T cells, and NK cells were significantly elevated in SRC-3 KD E0771 breast tumors compared to the control E0771 breast tumors (Fig. 7A–C). Additionally, Ifng levels were significantly increased in SRC-3 KD E0771 breast tumors compared to the control E0771 breast tumors (Fig. 7D). These results indicate that SRC-3 has a critical role in generating a tumor-enhancing immune microenvironment for breast cancer progression.

**Fig. 8:**
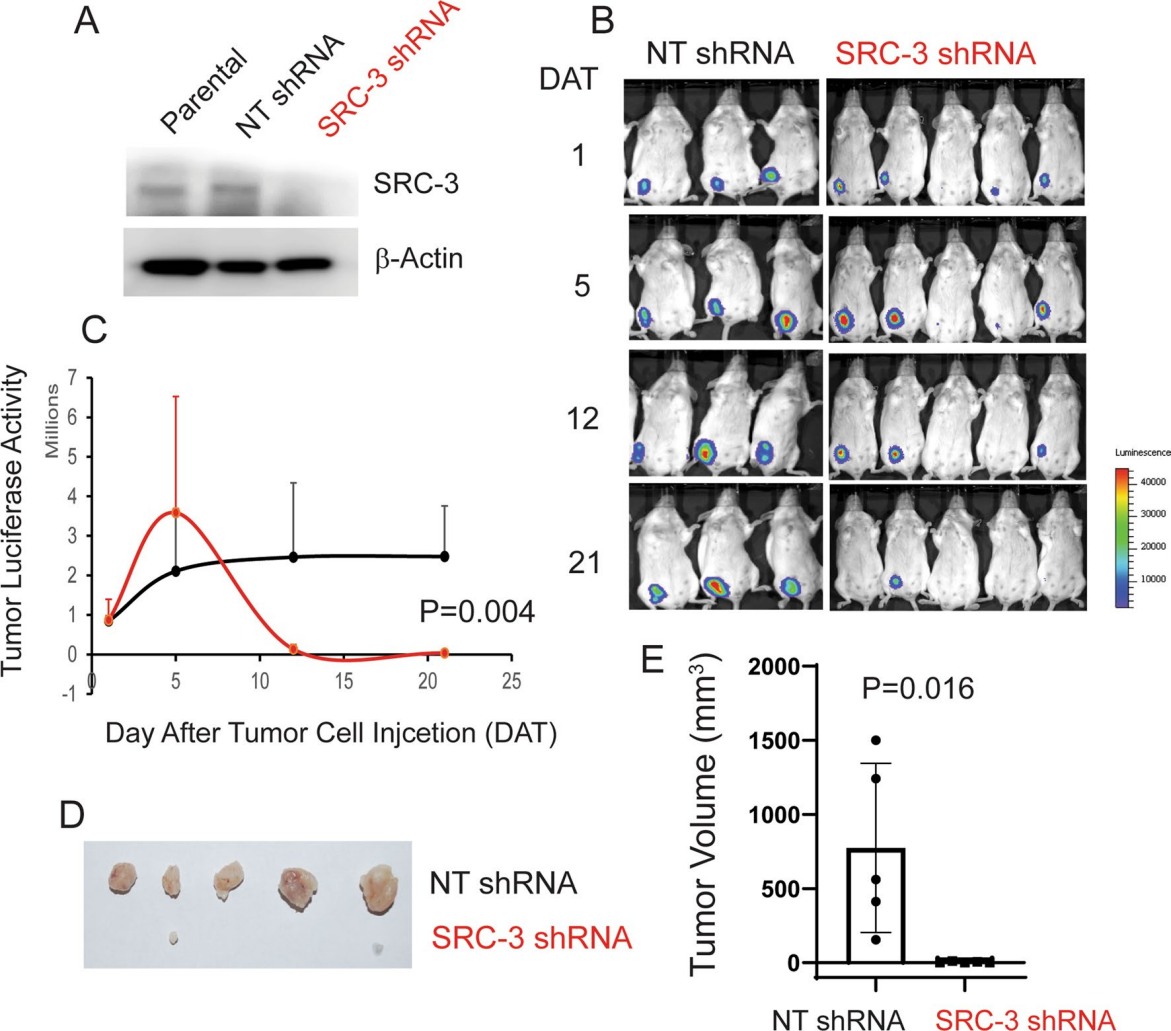
SRC-3 KD suppresses the 4T1 breast cancer progression in BALB/cJ female mice. **A** Western blot analysis of the SRC-3 levels in paternal 4T1 cells and 4T1 cells expressing a nontargeting (NT) shRNA and shRNA against mouse SRC-3. **B** Luciferase activity imaging analysis of the growth of luciferase-labeled SRC-3 KD 4T1 breast tumors and control luciferase-labeled 4T1 breast tumors expressing the nontargeting shRNA in BALB/cJ female mice. **C** Quantification of the luciferase activity of luciferase-labeled SRC-3 KD 4T1 and KD control 4T1 breast tumors in Panel B. **D** SRC-3 KD and control breast tumors harvested from tumor-bearing BALB/cJ mice on the 21st day after the injection of cancer cells. **E** Quantification of the volume of SRC-3 KD and control breast tumors in Panel D. Tumor volume was calculated as 0.5 x length × width × width

We also determined the effect of SRC-3 KD on 4T1 breast tumor progression in syngeneic immune-intact BALB/cJ female mice. SRC-3 shRNA significantly reduced SRC-3 protein levels in 4T1 cells compared to NT shRNA control cells (Fig. 8A). SRC-3 KD effectively inhibited 4T1 breast tumor progression in BALB/cJ female mice compared to KD control 4T1 cancer cells (Fig. 8B and C). After the final luciferase activity analysis, we harvested SRC-3 KD and control 4T1 breast tumors and determined their volumes. The SRC-3 KD 4T1 breast tumor volume was significantly lower than that of control 4T1 breast tumors (Fig. 8D, E). Therefore, SRC-3 is required for 4T1 breast tumor progression in immune-intact BALB/cJ female mice.

### Low-dose SI-2 (2.5 mg/kg) treatment and SRC-3 KD did not suppress the growth of breast tumors in immune-deficiency mice

**Fig. 9:**
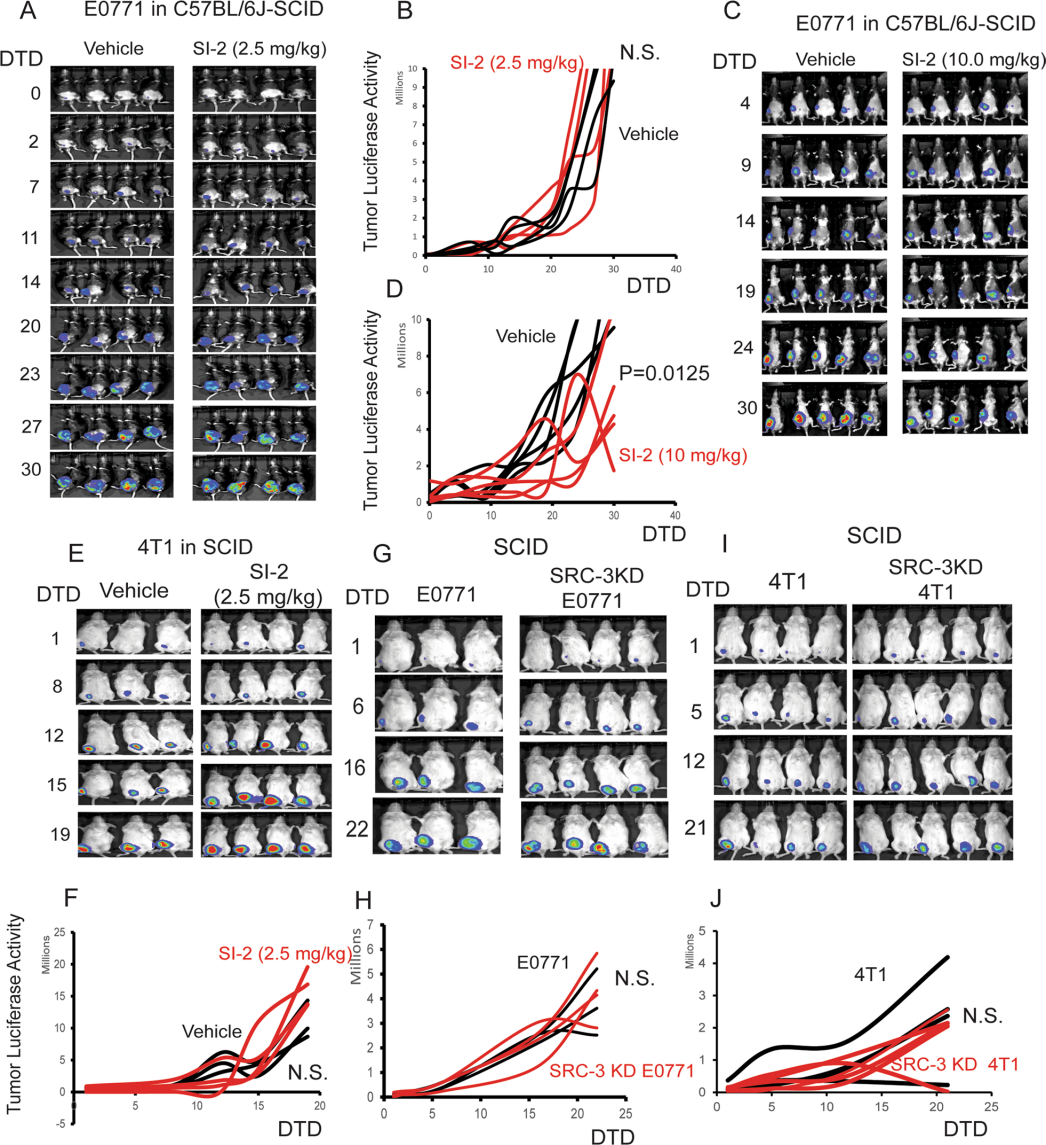
SI-2 treatment and SRC-3 KD do not suppress breast tumor growth in immune-deficient female mice. **A** Luciferase activity in E0771 breast tumors from C57BL/6J-SCID mice treated with SI-2 (2.5 mg/kg) or vehicle. **B** Quantification of luciferase activity in the E0771 breast tumors in Panel A is shown in the graph. **C** Luciferase activity in E0771 tumors from C57BL/6J-SCID mice treated with SI-2 (10 mg/kg) or vehicle. **D** Quantification of luciferase activity in the E0771 breast tumors in Panel C is shown in the graph. **E** 4T1 breast tumor luciferase activity in SCID mice treated with SI-2 (2.5 mg/kg) or vehicle. **F** Quantification of 4T1 breast tumor luciferase activity in Panel E is shown in the graph. **G** Luciferase activity in SRC-3 KD E0771 versus control KD E0771 breast tumors in SCID mice. **H** Quantification of luciferase activity in the SRC-3 KD E0771 versus control KD E0771 breast tumors in Panel G is shown in the graph. I Luciferase activity of SRC-3 KD versus control KD 4T1 breast tumors in SCID mice. **J** Quantification of the luciferase activity of SRC-3 KD versus control KD 4T1 cells in Panel I is shown in the graph. DTD, Drug Treatment Days. N.S., Non Specific

Although SI-2 treatment activated the host immune system to generate an antitumor immune response that suppressed E0771 breast cancer progression, we sought to obtain direct evidence supporting the hypothesis that SI-2-mediated suppression of E0771 breast cancer progression specifically requires host immune system function. Therefore, we employed C57BL/6J-SCID mice that carry the severe combined immunodeficiency mutation due to a spontaneous mutation of the PRKDC gene on the C57BL/6J background and thus lack functional T or B cells to answer this question [32]. In contrast to its effects on immune-intact C57BL/6J mice, SI-2 (2.5 mg/kg) treatment did not suppress E0771 tumor growth in C57BL/6J-SCID mice compared to vehicle-treated mice (Fig. 9A, B). However, a high dose of SI-2 (10 mg/kg) suppressed E0771 tumor growth in C57BL/6J-SCID mice compared to vehicle-treated mice (Fig. 9C, D).

To define the host immune effect of SI-2-mediated suppression of 4T1 breast tumor progression in immune-intact mice, 4T1 cells were orthotopically injected into female SCID mice (8 weeks old). SI-2 (2.5 mg/kg) treatment did not suppress the growth of 4T1 breast tumor progression in SCID mice compared to vehicle treatment, similar to E0771 breast tumors (Fig. 9E, F). Therefore, the low dose of SI-2 (2.5 mg/kg)-mediated breast tumor suppression required the host’s immune system.

SRC-3 KD suppressed E0771 breast tumor progression in immune-intact mice (Fig. 6). We determined whether breast tumor suppression mediated by SRC-3 KD occurred in immune-deficient mice. SRC-3 KD E0771 and KD control E0771 cells were orthotopically injected into SCID female mice. Compared to immune-intact mice, however, SRC-3 KD E0771 cells developed robustly into breast tumors in SCID mice compared to control E0771 cells (Fig. 9G, H). In contrast to immune-intact BALB/cJ female mice, SRC-3 KD did not suppress 4T1 breast tumor progression in SCID mice compared to NT shRNA control mice (Fig. 9I, J). Therefore, the host immune system is required to suppress SRC-3 KD breast tumor progression.

We conclude that a low dose of SI-2-mediated inhibition of breast tumor growth and suppression of SRC-3 KD breast tumor progression is a host immune system-dependent process.

### SI-2 treatment and SRC-3 KD changed the cytokine profile in breast tumors to suppress their progression

**Fig. 10:**
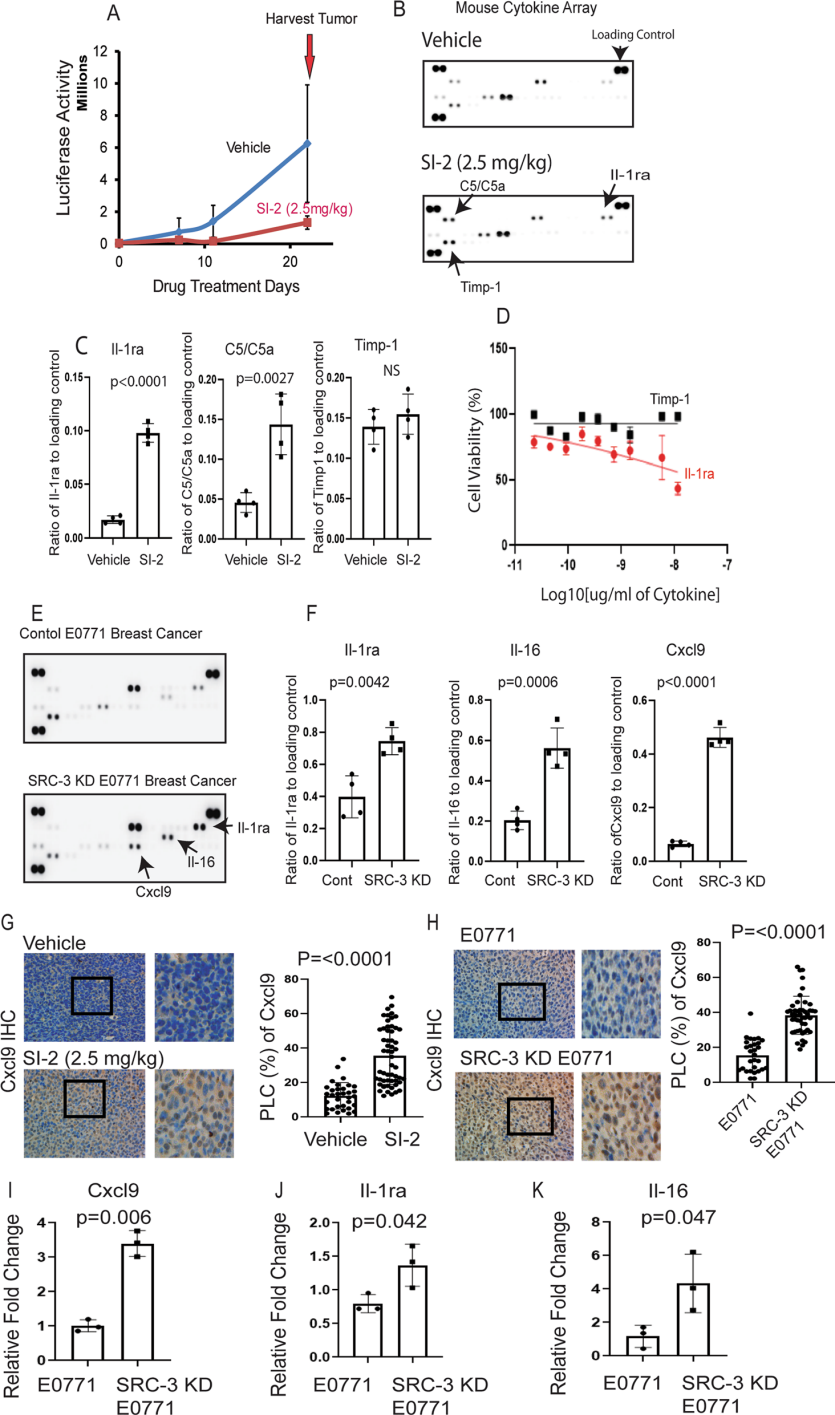
SI-2 treatment and SRC-3 KD change the cytokine profile in breast tumors to suppress tumor growth. **A** Luciferase activity in E0771 tumors from C57BL/6J mice treated with SI-2 (2.5 mg/kg) or vehicle. **B** Mouse cytokine array with cell lysates from E0771 tumors treated with SI-2 (2.5 mg/kg) or vehicle harvested from the mice shown in Panel A. **C** Quantification of the cytokine levels in Panel B is shown in the graph. **D** Inhibition of E0771 cell growth treated with different doses of IL-1RA but not Timp-1 compared to vehicle. The graph shows the quantification of the inhibition of cell growth by IL-1RA and Timp-1. **E** Mouse cytokine array with cell lysates from SRC-3 KD and control breast tumors. **F** Quantification of the cytokine levels in Panel E is shown in the graph. **G**–**H** Expression levels of Cxcl9 in SI-2-treated E0771 (**G**) and SRC-3 KD E0771 (**H**) breast tumor-bearing animals determined by IHC. Quantification of Cxcl9 levels in Panels g and h is shown in the graph. **I**–**K** The mRNA levels of Cxcl9 (**I**), Il-1ra (**J**), and Il-16 (**K**) in SRC-3 KD versus control KD E0771 cancer cells were determined

Cytokines mediate communication between immune and nonimmune cells to control innate and adaptive immune responses and suppress or enhance cancer progression [33]. To address whether SRC-3 inhibition changes the cytokine profile in breast tumors, E0771 tumors were harvested from tumor-bearing C57BL/6J mice on the 23rd day after SI-2 or vehicle treatment (Fig. 10A). Mouse cytokine profiling revealed that compared to vehicle controls, SI-2 treatment significantly increased the levels of complement component 5 (C5)/C5a and interleukin 1 receptor antagonist (Il-1ra) in E0771 breast tumors (Fig. 10B, C). Therefore, the elevation of Il-1ra by SI-2-mediated SRC-3 might suppress breast cancer progression because Il-1ra has tumor-suppressive activity [34]. Thus, we determined whether the increase in Il-1ra levels also suppressed the proliferation of E0771 breast cancer cells. E0771 cells were treated with different doses of recombinant human Il-1ra proteins. Compared to vehicle, Il-1ra effectively suppressed the proliferation of E0771 cells (Fig. 10D). As a control, we measured the effect of tissue metalloprotease inhibitor-1 (TIMP-1) on the proliferation of E0771 cells because SI-2 treatment did not significantly increase TIMP-1 expression in E0771 tumors compared to vehicle treatment (Fig. 10C). In contrast to Il-1ra, TIMP-1 did not suppress the proliferation of E0771 cells compared to vehicle-treated cells (Fig. 10D). Therefore, SI-2 increased Il-1ra levels in E0771 cancer cells to suppress their proliferation.

**Fig. 11:**
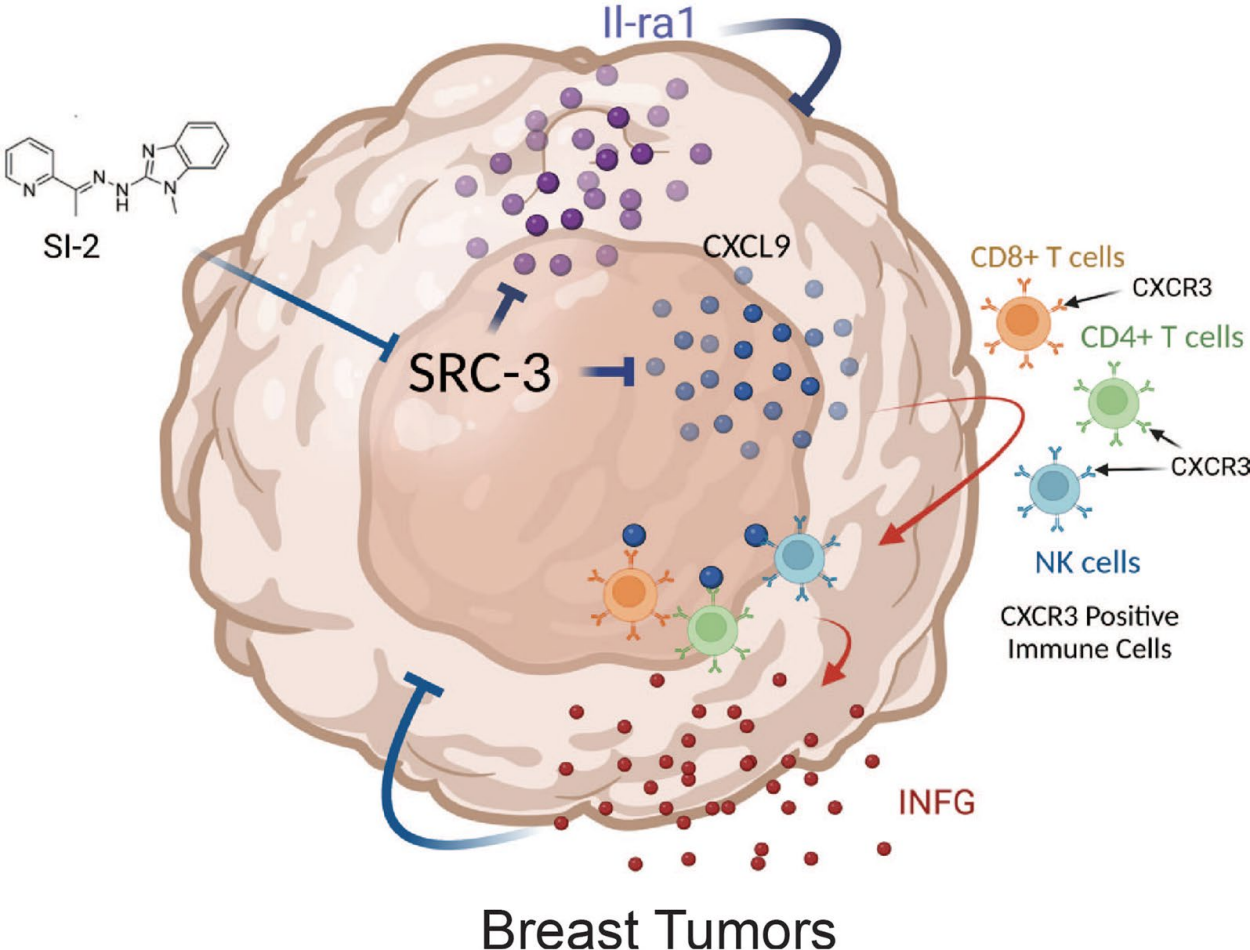
Model for SI-2 and SRC-3 KD-mediated inhibition of breast tumor progression by the tumor-suppressing immune environment. SI-2 treatment and SRC-3 KD elevated Cxcl9 levels in breast tumors and then recruited Cxcr3-positive cytotoxic CD8+, CD4 + T cells, and NK cells into the tumor. The elevated numbers of cytotoxic immune cells elevate Ifng, leading to reduced breast cancer progression. Additionally, SI-2 treatment and SRC-3 KD promoted Il-1ra expression to suppress the proliferation of breast tumors. The graphic was generated by BioRender

We next determined the cytokine profiles in SRC-3 KD and control E0771 breast tumors to address whether SRC-3 KD also changes the cytokine profile in breast tumors. Consistent with the cytokine array using SI-2-treated E0771 breast tumors, Il-1ra levels were increased in SRC-3 KD E0771 breast tumors compared to control breast tumors (Fig. 10E, F). Therefore, SRC-3 KD increased the expression of Il-1ra in breast tumors, leading to the suppression of E0771 breast cancer progression. Furthermore, Il-16 (2.7-fold) and C-X-C motif chemokine ligand 9 (Cxcl9, ninefold) levels were significantly increased in SRC-3 KD E0771 breast tumors compared to control breast tumors (Fig. 10E, F). IHC analysis revealed that Cxcl9 levels were elevated in SI-2-treated E0771 breast tumors (Fig. 10G) and SRC-3 KD E0771 breast tumors (Fig. 10H) compared with their control breast tumors. However, immune cells also produce Cxcl9, Il-1ra, and Il-16, while it is not clear whether SRC-3 KD breast tumors express these cytokines. To address this question, we cultured SRC-3 KD E0771 cells and control E0771 cells and then compared the levels of Cxcl9, Il-1ra, and Il-16 between them. RNA analysis revealed that SRC-3 KD enhanced the expression levels of Cxcl9, Il-1ra, and Il-16 in E0771 cells compared to control cells (Fig. 10I–K). Cxcl9 mainly regulates the recruitment of C-X-C motif chemokine receptor 3 (Cxc3R)-positive immune cells, such as CD4 + T cells, CD8 + T cells, natural killer (NK) cells, and macrophages [35]. Therefore, the targeting of SRC-3 by SI-2 and SRC-3 KD recruits Cxcr3-expressing cytotoxic immune cells, such as CD4 + , CD8 + , and CD56 + (NK cells), into breast cancers by increasing Cxcl9 and then generates an antitumor immune microenvironment to suppress breast cancer progression (Fig. 11) [36, 37].

## Discussion

SRC-3 gene amplification, high levels of SRCs, and hyperactivated SRC-3 increase the risk of breast cancer progression [7, 9, 38]. Therefore, we developed SI-2, a small-molecule inhibitor of SRC-3, as a novel breast cancer therapeutic agent, and SI-2 treatment suppressed the growth of MDA-MB-468 cells in immunodeficient mice by inhibiting SRC-3-mediated proliferation [11]. As shown in the present study, SI-2 treatment also reduced the progression of E0771 breast cancer in immune-intact syngeneic female mice by reducing SRC-3 levels in vitro and in vivo because SRC-3 plays an essential role in E0771 breast cancer progression [31]. Although SI-2 (2.5 mg/kg) effectively suppressed the progression of mouse breast tumors (E0771 and 4T1) in immune-intact mice, the same dose of SI-2 treatment did not effectively suppress E0771 and 4T1 tumor progression in immune-deficient mice. Therefore, the low dose of SI-2 effectively suppressed breast tumor progression using the host immune system. Blood cytokine profile analysis revealed that 10 mg/kg SI-2 treatment likely causes cytokine release syndrome in immune-intact mice due to high levels of blood cytokines compared to vehicle controls, leading to mouse illness [39]. Treatment with 5 mg/kg SI-2 did not cause mouse illness. However, compared to 2.5 mg/kg SI-2, 5 mg/kg SI-2 still significantly increased blood cytokine levels. We showed that 5 mg/kg SI-2 significantly reduced NK cell infiltration into E0771 breast tumors. Therefore, the high levels of blood cytokines might negatively impact NK cell function, reducing their antitumor activity at a dose of 5 mg/kg of SI-2 compared to 2.5 mg/kg of SI-2. Our previous study revealed that the inhibition of SRC-3 with low-dose SI-2 dramatically increased the proliferation of human peripheral blood mononuclear cells, bulk T cells, and bulk CD4 + cells [16]. Therefore, SRC-3 inhibition with a low dose of SI-2 should increase immune cell proliferation and elevate the numbers of tumor-infiltrating T cells to generate an immunosuppressive microenvironment compared to that observed in response to vehicle treatment.

SI-2 (2.5 mg/kg) effectively suppressed MDA-MB-468 breast cancer progression in SCID mice [11]. However, 2.5 mg/kg SI-2 did not suppress E0771 and 4T1 breast tumor progression in SCID mice, while 10 mg/kg SI-2 was able to suppress E0771 breast tumor progression in SCID mice. Although E0771, 4T1, and MDA-MB-468 cells are classified as TNBC lines [40–42], the mouse breast cancer cells (E0771 and 4T1) have a complex state of ER, PR, and HER2 expression compared to human TNBCs like the MDA-MB-468 line. The efficacy of chemotherapy is related to the status of hormone receptor expression [43]. In addition to this, broad genetic differences in TNBC and mouse syngeneic cancer cell lines likely underlie difference in response to anticancer agents. This is reflected by the fact that we observe that SI-2 has different suppressive effects on tumor growth for each of these lines in SCID mice. Therefore, the totality of differences in receptor expression and other genetic differences in these cell lines likely impact the differential outcomes seen for treatment with SI-2 on tumor growth in SCID mice.

SI-2 treatment significantly reduced the number of Foxp3 + Treg cells in E0771 breast tumors. Tregs have immunosuppressive activity by suppressing CD4 + T cell activation and proliferation through contact-dependent and contact-independent mechanisms [44] and impairing the proliferation of CD8 + CTLs by inducing apoptosis [45]. Additionally, Tregs suppress the cytotoxicity of CD56 + NK cells via the production of IL-10 [46]. Our recent study revealed that SRC-3 is highly enriched in Tregs in mice and humans and that SI-2 treatment decreased FOXP3 and CD25 levels in Treg cells, reducing their proliferation [16]. Additionally, SI-2-treated Treg cells did not suppress the proliferation of stimulated T cells. Therefore, SRC-3 inhibition shifted the immune response from a protumor to an antitumor phenotype by reducing the Treg population and increasing the TIL repertoire in E0771 tumors. In addition to Treg cells, Foxp3 is also expressed in cancer cells and promotes cancer growth and metastasis [47]. Our dual immunofluorescence staining revealed that SI-2 treatment also reduced the numbers of CD4-/Foxp3 + tumor cells in E0771 breast tumors. Therefore, we posit that SI-2 treatment might have a dual function in suppressing breast cancer progression by reducing the Treg population and blocking Foxp3 function in breast cancer cells.

A mouse cytokine array revealed that Cxcl9 levels were significantly increased in SRC-3 KD E0771 breast tumors and SI-2-treated E0771 breast tumors compared to control breast tumors. CXCL9 mainly regulates immune cell migration, differentiation, activation, and recruitment of CXCR3-expressing immune cells, such as cytotoxic lymphocytes (Th1-type CD4 + T cells and CD8 + T cells), natural killer cells, and macrophages [35, 36]. Therefore, SI-2-mediated SRC-3 inhibition increased the levels of Cxcl9 in breast tumors to recruit cytotoxic immune cells. Activated CD4 + T cells secrete interleukin (IL)-2, which directly activates CD8 + cytotoxic T lymphocytes (CTLs) expressing the high-affinity IL-2 receptor α subunit (CD25) to drive their effector function, differentiation, and proliferation [48]. Additionally, Th1-type CD4 + T cells exert direct antitumor effects by secreting IFNG and TNFα [49]. CD8 + CTLs are critical in immune defenses against tumors by secreting cytokines, primarily TNF-α and IFNG [50, 51]. CD8 + CTLs produce cytotoxic granules containing perforin and granzymes to kill cancer cells by attacking the membrane complex and inducing apoptosis [52]. CD56 is considered a key marker for natural killer (NK) cells [53]. However, CD56 is expressed not only in NK cells but also in many other immune cells, including αβT cells, γδT cells, dendritic cells, and monocytes [24, 54, 55]. Interestingly, these CD56-expressing cell types also perform vital immunostimulatory effector functions. Therefore, the increase in the cytotoxic immune cell population by SRC-3 inhibition is also expected to contribute to antitumor immunity in E0771 tumors to suppress their progression.

IL-1β-mediated activation of the NOD-like receptor (NLR) inflammasome plays a vital role in developing various cancers, and the blockade of IL-1β using recombinant IL-1RA significantly decreases tumor progression [56–58]. For example, mouse Il-1ra (5 mg/kg) treatment significantly reduced the growth of E0771 breast tumors in C57BL/6J mice compared to vehicle-treated mice [59]. Therefore, SRC-3 inhibition suppresses NLR-mediated inflammasome pathways in E0771 tumors by preventing Il-1β function by increasing Il-1ra levels and suppressing breast cancer proliferation.

## Conclusions

Previous studies focused on the role of SRC-3 in primary breast tumors have defined how targeting SRC-3 affects cell autonomous growth pathways within breast cancer cells. However, using a syngeneic immune-intact mouse model of breast cancer, we show that SRC-3 is also a critical immunomodulator that generates a protumor immune microenvironment during breast cancer progression. Therefore, SRC-3 inhibition activates the Cxcl9/Cxcr3 axis, causing an antitumor immune microenvironment by recruiting cytotoxic immune cells into breast tumors.

## Supplementary Information

**Additional file 1**.** Fig. S1**: Low doses of SI-2 exhibit better tumor-suppressive activity than high doses of SI-2.** A** Reduction in luciferase activity in E0771 tumors in B6 albino mice by SI-2 (2.5 mg/kg and 5 mg/kg) treatment compared with vehicle treatment. Quantification of luciferase activity in the E0771 breast tumors shown in Panel A. ** B–D**. Numbers of CD4+ T cells (**B**), CD8+ T cells (**C**), and CD56+ NK cells (**D**) in E0771 breast tumors treated with vehicle or 2.5 or 5 mg/kg SI-2. Quantifications of the CD4+ T cells, CD8+ T cells, and CD56+ NK cells levels are shown in the graph.

**Additional file 2**.** Fig. S2**: List of blood cytokines in C57BL/6J female mice treated with vehicle or 2.5, 5, or 10 mg/kg SI-2 twice a day for 7 days.

## Data Availability

The datasets used and/or analyzed during the current study are available from the corresponding author upon reasonable request.

## Abbreviations

CCL: Chemokine (C-C motif) ligand
Cxcl9: C-X-C motif chemokine ligand 9
Cxcr3: C-X-C motif chemokine receptor 3
ERα: Estrogen receptor alpha
HER2: Human epidermal growth factor receptor 2
Ifng: Interferon gamma
Il-16: Interleukin 16
Il-1ra: Interleukin-1 receptor antagonist
IVIS: In vivo imaging system
KD: Knock down
MCP-1: Monocyte chemoattractant protein-1
NK: Natural killer
PBS: Phosphate-buffered saline
PR: Progesterone receptor
SCID: Severe combined immunodeficiency
SRC-3: Steroid receptor coactivator-3
TIME: Tumor immune microenvironment
TIMP-1: Tissue inhibitor matrix metalloproteinase 1
