# Supplementary figures and images for: Steroid receptor coactivator-3 inhibition generates breast cancer antitumor immune microenvironment
Source: Breast Cancer Res. 2022 Oct 31;24:73. doi: 10.1186/s13058-022-01568-2 (PMC9620627; doi:10.1186/s13058-022-01568-2)

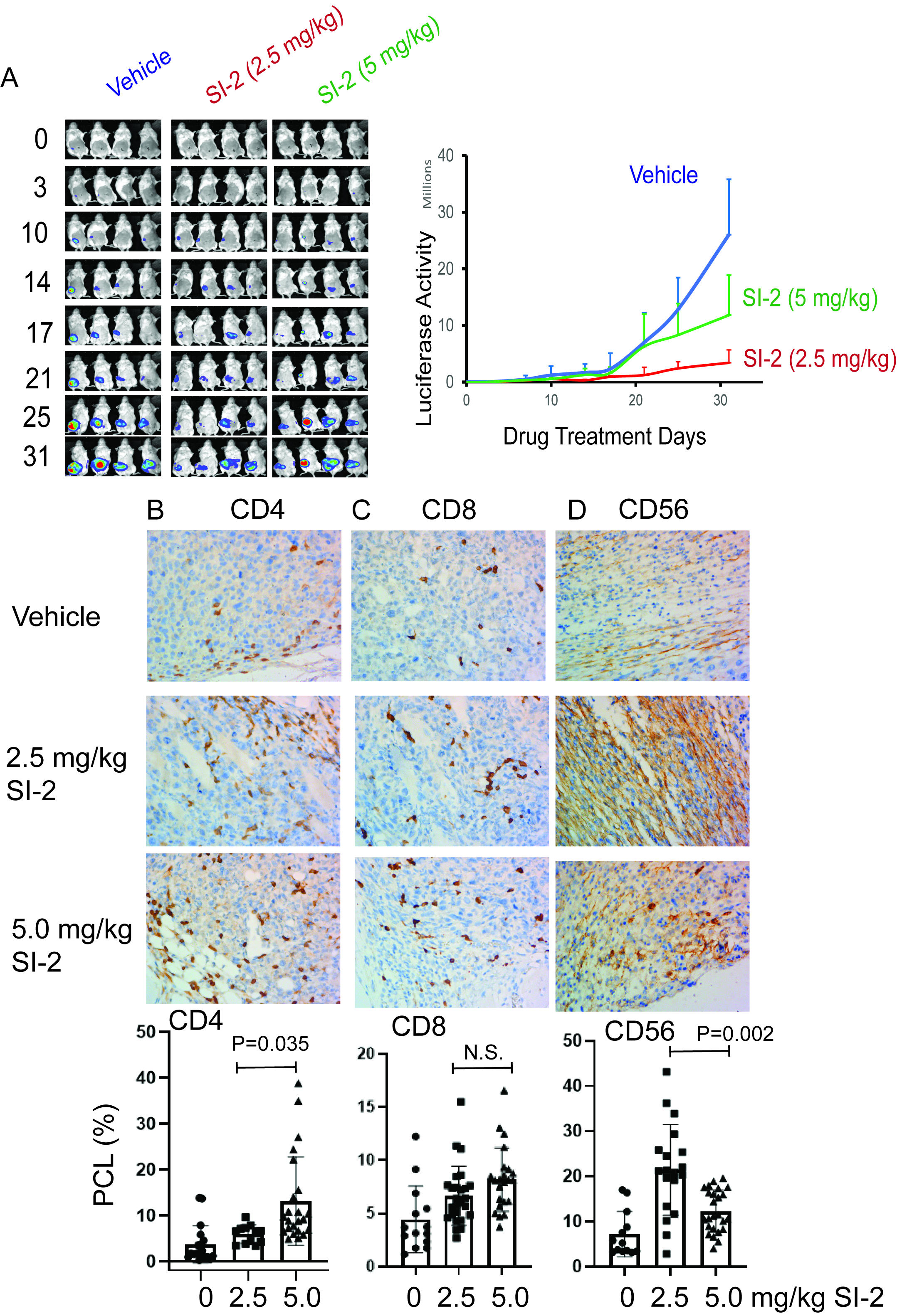

Supplement: Supplementary file 1 — Additional file 1. Fig. S1: Low doses of SI-2 exhibit better tumor-suppressive activity than high doses of SI-2. A Reduction in luciferase activity in E0771 tumors in B6 albino mice by SI-2 (2.5 mg/kg and 5 mg/kg) treatment compared with vehicle treatment. Quantification of luciferase activity in the E0771 breast tumors shown in Panel A. B–D. Numbers of CD4+ T cells (B), CD8+ T cells (C), and CD56+ NK cells (D) in E0771 breast tumors treated with vehicle or 2.5 or 5 mg/kg SI-2. Quantifications of the CD4+ T cells, CD8+ T cells, and CD56+ NK cells levels are shown in the graph. [file 13058_2022_1568_MOESM1_ESM.tif]

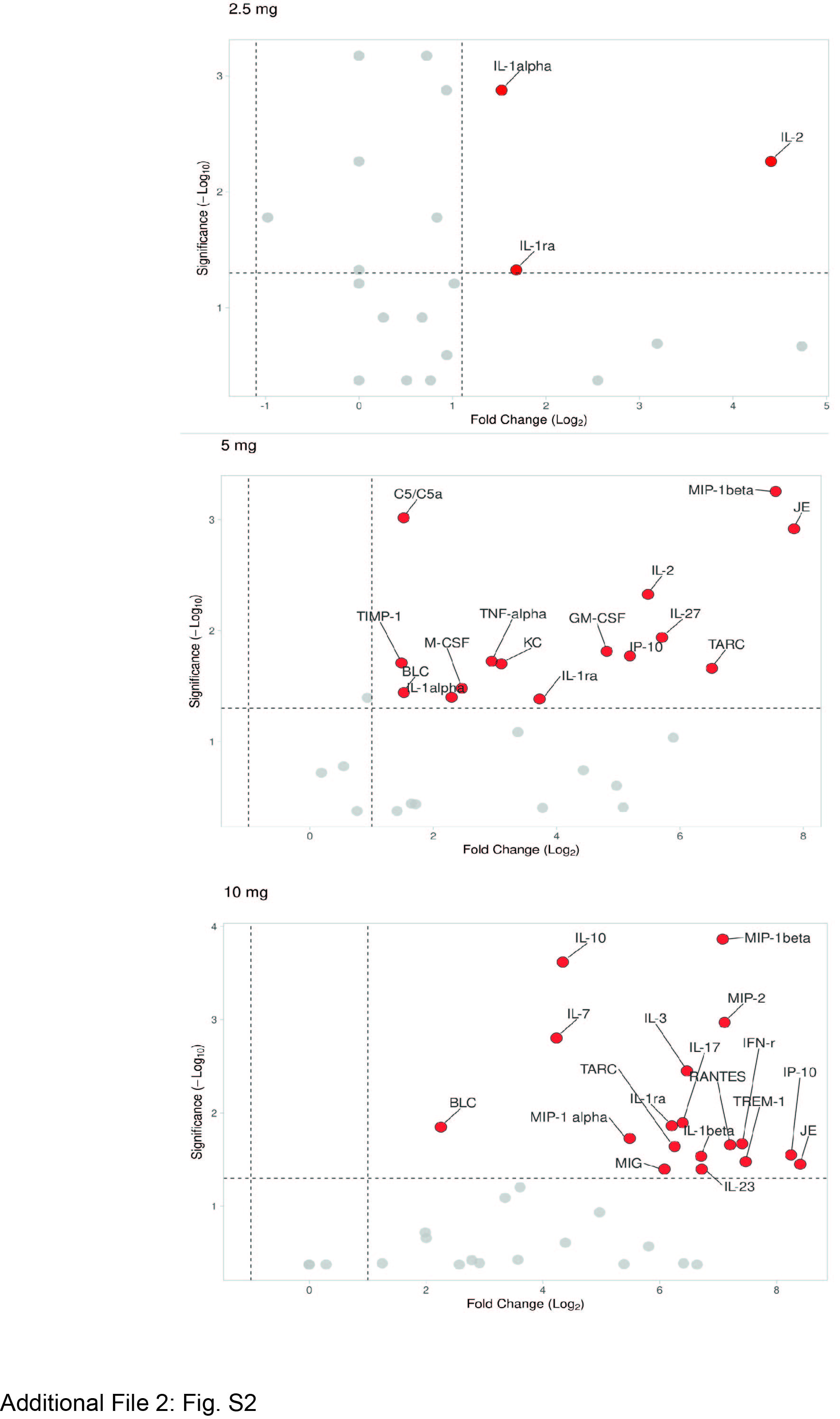

Supplement: Supplementary file 2 — Additional file 2. Fig. S2: List of blood cytokines in C57BL/6J female mice treated with vehicle or 2.5, 5, or 10 mg/kg SI-2 twice a day for 7 days. [file 13058_2022_1568_MOESM2_ESM.jpg]
